# The stage of soil development modulates rhizosphere effect along a High Arctic desert chronosequence

**DOI:** 10.1038/s41396-017-0026-4

**Published:** 2018-01-15

**Authors:** Francesca Mapelli, Ramona Marasco, Marco Fusi, Barbara Scaglia, George Tsiamis, Eleonora Rolli, Stilianos Fodelianakis, Kostas Bourtzis, Stefano Ventura, Fulvia Tambone, Fabrizio Adani, Sara Borin, Daniele Daffonchio

**Affiliations:** 1 Department of Food, Environmental and Nutritional Sciences, University of Milan, Milan, 20133 Italy; 2 King Abdullah University of Science and Technology (KAUST), Biological and Environmental Sciences and Engineering Division (BESE), Thuwal, 23955-6900 Saudi Arabia; 3 Department of Agricultural and Environmental Sciences—Production, Landscape, Agroenergy, University of Milan, Milan, 20133 Italy; 4 Department of Environmental and Natural Resources Management, University of Patras, Agrinio, 30100 Greece; 5 Institute of Ecosystem Study, CNR, Sesto Fiorentino, 50019 Italy

## Abstract

In mature soils, plant species and soil type determine the selection of root microbiota. Which of these two factors drives rhizosphere selection in barren substrates of developing desert soils has, however, not yet been established. Chronosequences of glacier forelands provide ideal natural environments to identify primary rhizosphere selection factors along the changing edaphic conditions of a developing soil. Here, we analyze changes in bacterial diversity in bulk soils and rhizospheres of a pioneer plant across a High Arctic glacier chronosequence. We show that the developmental stage of soil strongly modulates rhizosphere community assembly, even though plant-induced selection buffers the effect of changing edaphic factors. Bulk and rhizosphere soils host distinct bacterial communities that differentially vary along the chronosequence. Cation exchange capacity, exchangeable potassium, and metabolite concentration in the soil account for the rhizosphere bacterial diversity. Although the soil fraction (bulk soil and rhizosphere) explains up to 17.2% of the variation in bacterial microbiota, the soil developmental stage explains up to 47.7% of this variation. In addition, the operational taxonomic unit (OTU) co-occurrence network of the rhizosphere, whose complexity increases along the chronosequence, is loosely structured in barren compared with mature soils, corroborating our hypothesis that soil development tunes the rhizosphere effect.

## Introduction

Soil type and edaphic factors shape the assembly of microbiota in mature soil and primarily contribute to the availability of microorganisms to be recruited by plant roots in the rhizosphere [1–3]. Soil type plays an important role in agricultural ecosystems, whereas plant species has a stronger impact in natural environments [2]. In glacier foreland chronosequences, different soil developmental stages occur on a limited spatial and temporal scale [4, 5]. Although primary successions [4, 5], soil [6–13], and rhizosphere communities of pioneer plants [12, 14] have been previously described, microbiota interactions in the rhizosphere under different soil developmental phases have rarely been examined [15].

It has been shown that soil microbiota changes with time since deglaciation [10, 11], therefore plant roots are exposed to variable microbial taxa during the process of soil development. We hypothesize that the recruitment of the rhizosphere microbiota by pioneer plants and the bacterial network topology of these microbiota are driven by the changing environmental conditions of the different soil developmental stages across the chronosequence of soil formation.

**Fig. 1:**
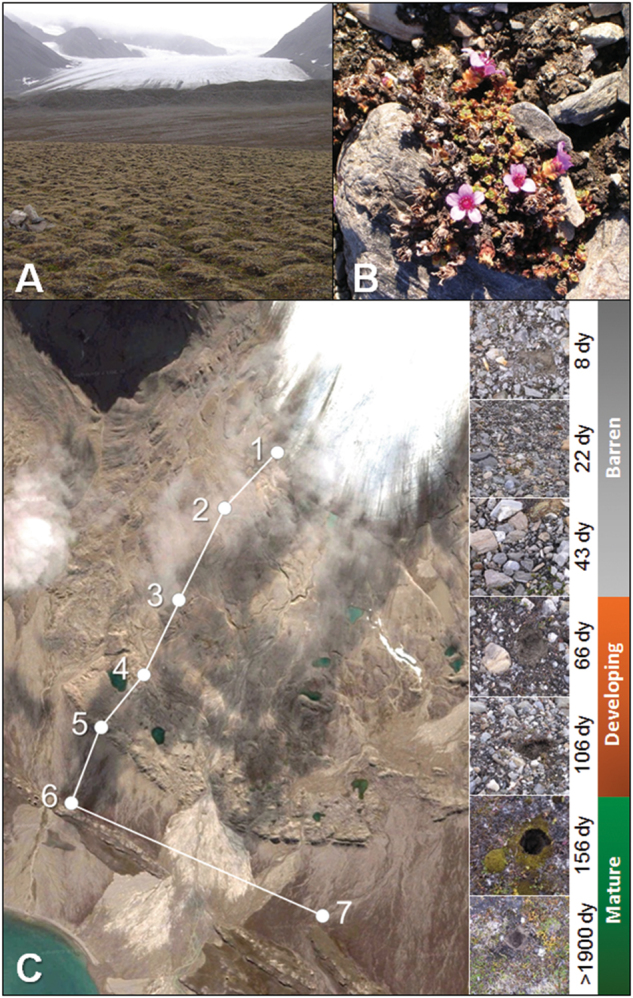
The Midtre Lovénbreen glacier chronosequence. **a** Overview of the Midtre Lovénbreen glacier moraine (early September 2006), showing the gradual shift from the barren to mature substrate. **b** Example of the *S. oppositifolia* plants collected across the Midtre Lovénbreen chronosequence. **c** Scheme of the chronosequence described by Hodkinson and co-authors [4], indicating the position of the seven dated sites. Pictures on the right side of the image were taken during sample collection and show the macroscopic diversity of the soil surface at increasing times since deglaciation. Further details on soil characteristics and type and level of plant coverage on soil can be found in Hodkinson et al. [4]

To test our hypothesis, we selected the pioneer plant species *Saxifraga oppositifolia*, a colonizer of all the seven sites of the Midtre Lovénbreen glacial chronosequence on the Svalbard archipelago across a 1.7 km transect revealing about 2000 years of soil development with increasing levels of nutrient availability, soil fertility, and plant colonization (Fig. 1; [4, 10, 11]). We examined bacterial community diversity and network topology in the bulk soil and in the *S. oppositifolia* rhizosphere of the different soil developmental stages occurring across the chronosequence. Chronosequences provide a solid framework to pursue this aim, consisting of successive soil developmental stages that can be identified on glacier moraines, where the increasing distance from glacier edge corresponds to the increase in time since deglaciation, soil structuring, and ecosystem development as a consequence of primary colonization by microbes and plants occurring after ice melting [5]

## Materials and methods

### Study area and sampling

All the samples were collected in the moraine of the Midtre Lovénbreen glacier (78°53’N), Ny Ålesund, Svalbard, in early September 2006 (Fig. 1) along the Hodkinson proglacial chronosequence [4]. Characterization and dating of the seven sites in the linear transect of the chronosequence were determined by photographic records and radiocarbon analyses, and the site position was referenced by GPS coordinates and ground stakes [4]. At the time of sampling, sites 1–7 corresponded to a time of exposure after ice melting of 8, 22, 43, 66, 106, 156, and >1900 years, respectively. All the sites were characterized by a specific and dynamic floristic composition frequency and the ground cover was changing along the sites, with a constant layer of *Cyanobacteria* covering the soil surface along the entire chronosequence [4]. The surface of sites 6 and 7 showed a full vegetation coverage (Fig. 1; [4, 16]). *S. oppositifolia*, a pioneer vascular plant typical of arctic and alpine regions, was described as the most abundant vascular plant throughout the chronosequence at each sampling site [4]. Thus, we chose *S. oppositifolia* as a representative plant species for our study (Fig. 1b). In each site, three isolated *S. oppositifolia* plants of the same size were selected, based on visual observation. The plants recovered were grouped according to the ‘soil developmental stage’ (levels: ‘barren’, ‘developing’, and ‘mature’) as defined in the following paragraph according to the rationale explained in the Results and Discussion section. All the sites were sampled on the same day. In order to aseptically collect the rhizosphere fraction, the entire plant was removed from the soil and gently shaken to remove the soil not tightly attached to the root. Within 6 h of sampling, the root systems were separated from the plant and collected in sterile plastic bags. The pull and shake method was first applied to separate the rhizosphere soil particles spontaneously detached from the roots. The remaining roots were placed in sterile tubes containing 9 mL of physiological solution (9 g/L NaCl) to obtain the rhizosphere interface, which is the soil that is tightly attached to the root rhizoplane. The tubes were vortexed for 5 min and centrifuged at 4000 rpm for 5 min. The supernatant was discarded and the remaining soil fraction was merged with the rhizosphere soil previously detached to be used as the rhizosphere fraction. From each site, three bulk soils not in contact with the root system of *S. oppositifolia* or other visible plants and located at 50–100 cm from each sampled plant were also aseptically collected at 3–5 cm depth after removing the 1–2 cm thick moss carpet (comprising moss rhizosphere). Both bulk soil and rhizosphere samples were stored at −20 °C for molecular analysis.

### Soil analyses and characterization

Soil pH, total nitrogen (NTK), total (TOC), and dissolved organic carbon (DOC), cation exchange capacity (CEC), exchangeable cations (Ca_exc_, Mg_exc_, K_exc_, Na_exc_), total Ca, Mg, K, Na, Mn, P content, and soil respiration were determined as previously reported [17]. Dissolved organic matter (DOM) extraction, chemical, and metabolite characterizations were performed as previously reported [18, 19]. Detailed information on the materials and methods is presented in Supplementary Method 1.

We assessed the similarity of the recovered soil samples by canonical analysis of principal coordinates (CAP) [20] of their physicochemical properties (Supplementary Table 1A), as described in Supplementary Method 4, clustering the soil samples according to their belonging to the ‘soil developmental stage’ (levels: ‘barren’, ‘developing’, and ‘mature’; defined as explained in the Results and Discussion section).

### Automated ribosomal intergenic spacer analysis, PhyloChip analysis, and 16S rRNA gene sequencing

Total DNA was extracted from 0.5 g of each replicate soil sample with the PowerSoil® DNA Isolation Kit (MoBio Inc., CA, USA) following the manufacturer’s instructions and stored at −20 °C until further processing. DNA was quantified using a PowerWave HT Microplate Spectrophotometer (BioTek). The automated ribosomal intergenic spacer analysis fingerprinting of 16S–23S ribosomal RNA (rRNA) was performed as previously reported [21]. Near-full-length 16S rRNA gene amplification for the PhyloChip analysis was carried out using universal 16S rRNA primers for bacteria (27F and 1492R) [22] as described in Supplementary Method 2. High-throughput Illumina sequencing was performed on the V4–V5 hypervariable regions of the 16S rRNA gene. The full description of the materials and methods for 16S rRNA gene sequencing, including the workflow scripts and commands, is presented in Supplementary Method 3. Raw sequences were deposited in the ENA European Read Archive under accession number PRJEB12640.

### Diversity, phylogenetic, and statistical analyses

A principal coordinate analysis was used to assess the phylogenetic β-diversity based on the Bray–Curtis matrix of the 16S rRNA gene Illumina sequence data set. Significant clustering among sample groups was tested by permutational multivariate analysis of variance (PERMANOVA) considering both the ‘fraction’ (levels: ‘bulk soil’, ‘rhizosphere’) and ‘soil developmental stage’ (levels: ‘barren’, ‘developing’, and ‘mature’; defined as explained in the Results and Discussion section) as orthogonal and fixed factors.

For phylogenetic β-diversity analyses, we calculated the β-nearest taxon index (βNTI) for each soil fraction and between every consecutive soil stage (see below) as described elsewhere [23]. βΝΤΙ values smaller than −2 indicate homogenous selection, values between −2 and 2 indicate stochasticity, and values larger than 2 indicate variable selection. All the statistical tests and the diversity indices were performed with PRIMER v. 6.1, PERMANOVA+ for PRIMER routines [24]. An analysis of covariance was applied to test whether the rate of decay of community similarity (Bray–Curtis) along the soil developmental stages was different between the rhizosphere and the bulk soil. Linear discriminant analysis (LEfSe, www.huttenhower.sph.harvard.edu/galaxy/) was applied on the operational taxonomic unit (OTU) table according to the method of Segata and co-workers [25], to identify bacterial taxa that could be detected as discriminant among the soil developmental stage groups (for more details see Supplementary Method 4). Significant correlation of bacterial communities based on the Illumina 16S rRNA gene-based data set with physicochemical data and metabolite concentrations were assessed with distance-based multivariate analysis for a linear model (DistLM, Supplementary Method 5). Co-occurrence network analysis was performed on the Illumina 16S rRNA gene-based data set for both bulk soil and rhizosphere along the soil developmental stages by using nine, six and six replicates for the ‘barren’, ‘developing’, and ‘mature’ soil stages, respectively [26–28], as described in detail in Supplementary Method 6.

## Results and Discussion

### Specific bacterial communities are selected in the *S. oppositifolia* rhizosphere and shifted along the Midtre Lovénbreen chronosequence according to the soil developmental stage

**Fig. 2:**
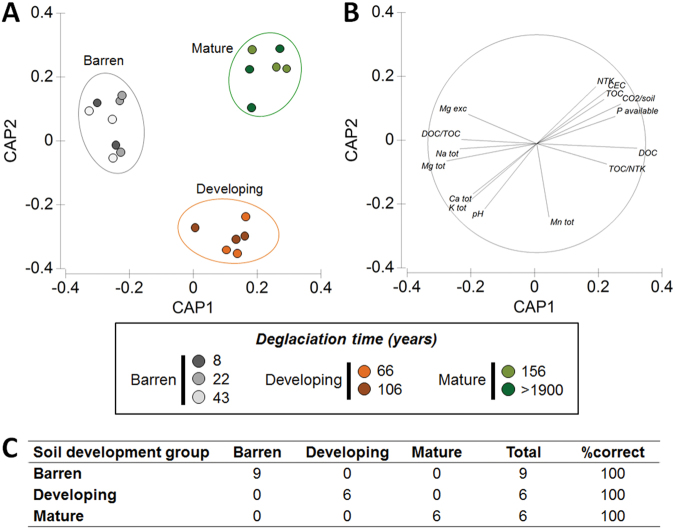
Definition of the soil developmental stage groups of the seven sites of the Midtre Lovénbreen chronosequence. **a** Canonical analysis of principal coordinates (CAP) of the soil physicochemical data from the seven Midtre Lovénbreen sites and their **b** Pearson correlations (*r* ≥ 0.8) of original environmental variables with the canonical axes. **c** CAP cross-validation (choice of m: 9; no. of permutations used: 9999; delta_1^2: 0.96115, *p* = 0.0003). The data set is described in Supplementary Table 1. NTK total nitrogen, TOC total organic carbon, CEC cation exchange capacity, exc exchangeable, tot total, P available, K, Ca, Mg, Na, Mn concentrations are expressed as mg/kg DM; NTK and TOC concentration are expressed as g/kg DM; DOC concentration is expressed as mg/kg DM

As our hypothesis is that the developmental state of the soil rather than the time since deglaciation is a key factor in determining the assembly of the rhizosphere communities, we aimed to use the soil developmental stages as the categorical explanatory variable of the analyses. The CAP of physicochemical soil analysis (Supplementary Table 1), detected three significantly different developmental stage groups (PERMANOVA, *F*_2,19_ = 7.03; *p* = 0.0001; Fig. 2) which diversity is explained by different physicochemical parameters (Supplementary Table 1C-D). We defined the three groups as ‘barren’ (8, 22, and 43 years old soils under the first phases of development), ‘developing’ (66 and 106 years old soils under an intermediate phase of development), and ‘mature’ (156 and >1900 years old mature soil). According to this characterization, the three groups of soil developmental stages included nine replicates for samples belonging to the ‘barren’ soil stage, six replicates belonging to the ‘developing’ soil stage, and six belonging to the ‘mature’ soil stage, both for the rhizosphere and bulk fractions. The nutrient concentrations, fertility-related soil properties, and TOC increased along the chronosequence and the highest values were detected in the ‘mature’ soils (Supplementary Table 1A). This is in agreement with the plant and soil characterization described by Hodkinson et al. [4] and with the site spatial pattern, used as space-for-time substitution in chronosequence and ecosystem development studies [5]. Despite the increase of TOC along the chronosequence caused by the time of vegetation presence [4], the main factors explaining the differences among the soil developmental stages change along the chronosequence (Supplementary Table 1C-D).

**Fig. 3:**
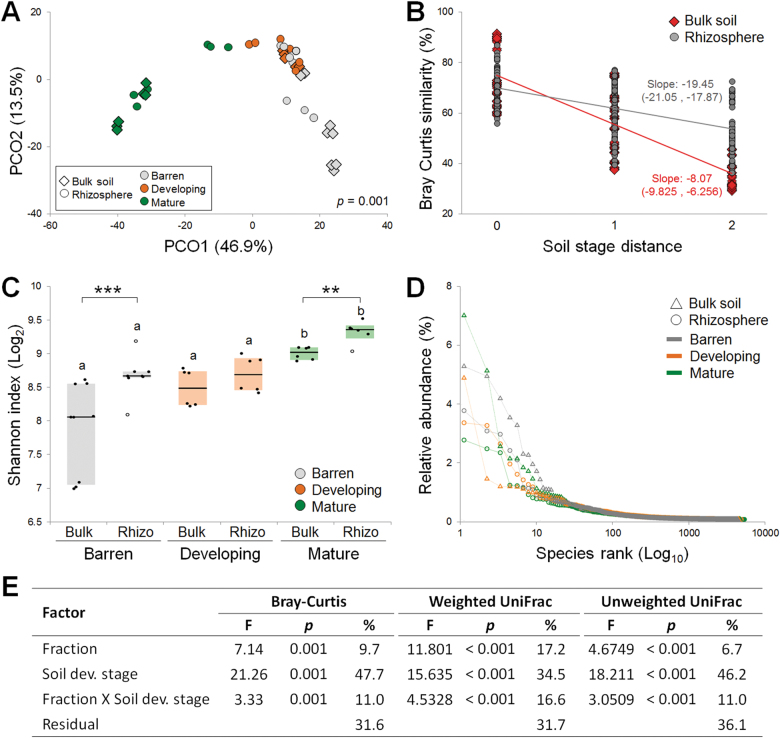
Bacterial community structure (alpha- and beta-diversity) associated with the rhizosphere of *S. oppositifolia* and bulk soils along the Midtre Lovénbreen chronosequence. **a** Principal coordinates analysis showing the clustering of bacterial communities according to soil fractions and developmental stage. **b** Distance decay analysis showing the trend of bacterial community similarity in rhizosphere and bulk samples according to soil stage difference across the chronosequence; mean slopes are given and their 95% interval of confidence are indicated in brackets. In the x axis '0' indicates the distance between samples belonging to the sam developmental stage; '1' indicates distamce between samples belonging to 'barren vs developing' and 'developing vs mature'; '2' indicates distance between samples belonging to 'barren vs mature'. **c** Shannon index box plot. The letters indicate statistical differences among the soil developmental stages in the same soil fraction (see Supplementary Table 3); asterisks (as per Kruskall–Wallis tests ** for *p* < 0.01 and *** for *p* < 0.001) indicate significant differences between the two fractions (rhizosphere and bulk soil) within the three soil developmental stages. **d** Dominance percentage (%) within bulk soil and rhizosphere OTUs of ‘barren’, ‘developing’, and ‘mature’ soil stages. **e** Factors determining the bacterial community variation using PERMANOVA (999 permutations) of Bray–Curtis dissimilarity and weighted and unweighted UniFrac distances for the indicated factors. In each analysis, F, the *p*-value and the percentage of variation (%) explained by each factor refers to the total variance reported

PhyloChip analysis of 16S rRNA gene and fingerprinting analysis of the 16S–23S rRNA ribosomal spacers showed that bacterial communities in the bulk soil and *S. oppositifolia* rhizosphere were significantly different (Supplementary Figure 1 and 2). Analysis of an Illumina 16S rRNA gene sequence data set further revealed a significantly strong interaction among the factors soil fraction and soil developmental stage (PERMANOVA; *F*_2,35_ = 3.33; *p* = 0.001, Figs. 3a, e and Supplementary Table 2 for post-hoc test analysis).

Distance decay analysis indicated a significant decrease in community similarity among distant samples across the soil developmental stages (*R* ^2^ = 0.63 and 0.29 for bulk soil and rhizosphere, respectively, *n* = 190, *p* < 0.001 for both correlations, Fig. 3b) with a higher rate of decay in bulk soils than in rhizospheres (analysis of covariance, *p* < 0.001, mean slope values of −19.46 and −8.07 and slope 95% confidence intervals of (−21.05, −17.87) and (−9.83, −6.26), for the bulk soil and rhizosphere, respectively). In both the bulk soil and rhizosphere fractions, the Shannon diversity index was significantly higher in the ‘mature’ soil (Kruskall–Wallis test, *H*_c_ = 12.87, *p* = 0.002; Fig. 3c), and higher in the rhizosphere than in the bulk soils at each developmental stage (except for the ‘developing’ soil, Supplementary Table 3). Rhizosphere soils hosted higher numbers of rare (<0.5% in relative abundance) bacterial OTUs (Fig. 3d). The observed higher diversity in the rhizosphere than in the bulk soil differs from common observations in agricultural soils [3], but is in agreement with observations in other glacier [14] and soil reclamation [29] chronosequences. This trend was similar to findings in previous studies from low and high arcto-climate zones, where the rhizosphere samples had highest richness and diversity [30, 31]. Similarly, other studies on glacier forefield or desert soils reported higher bacterial diversity and richness values in the rhizospheres than in the corresponding bulk soils [32–34]. This may indicate a plant nurturing effect on bacterial communities that becomes significantly more relevant in soils with challenging conditions, such as arctic soil. Although we cannot exclude that the PCR-based approach we used may have undersampled certain taxa in the bulk soil [35], we speculate that in glacier moraine and desert soils, plant exudates may represent remarkably diverse additions to the poor carbon source landscape of the barren bulk soil that may enhance diversity in the rhizosphere (Dümig et al. 2012; [36]).

The βNTI index increased across the three soil developmental stages for both bulk soil and the rhizosphere bacterial communities (7.69 ≤ βNTI ≤ 24.46; Supplementary Table 4); this is a sign of increasingly strong heterogeneous selection as the soil developed from ‘barren’ to ‘mature’. An increasing heterogeneous selection indicates a shift toward a more heterogeneous environment. This can be explained by the progressive complexity of the soil from the barren to the mature developmental stage. The increase of TOC, NTK, and CEC and decrease of the DOC/TOC ratio along the three soil developmental stages of the chronosequence (see also [4]), support a progressive accumulation of more heterogeneous compounds during the pedogenesis process.

Quantification of the contributions of individual factors to the observed bacterial community variations, determined by PERMANOVA of the Bray–Curtis dissimilarity and unweighted and weighted UniFrac distance metrics (Fig. 3e), showed that the ‘soil developmental stage’ factor is a major determinant that explains 34.5–47.7% of the observed bacterial community variation, followed by the ‘fraction’ factor (bulk soil or rhizosphere, 6.7–17.2%), and their interaction (11–16.6%). A significant effect of soil type has recently been measured in the root microbiota of the alpine plant *Arabis alpina* [37]. Our measures indicate that the selective pressure imposed by the conditions of the heterogeneous soil along the chronosequence is a strong driver of community phylogeny, irrespective of the buffering effect of the rhizosphere.

**Fig. 4:**
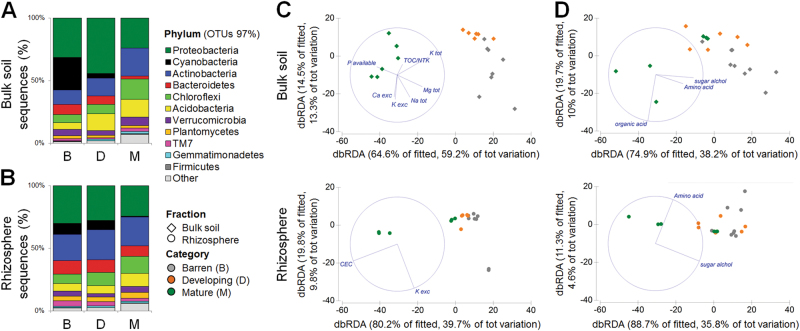
Taxonomy and correlation of bacterial diversity with soil properties across the Midtre Lovénbreen chronosequence. Bar charts analysis showing the relative abundance of the main phyla associated with bulk soils **a** and *S. oppositifolia* rhizospheres **b**. Distance-Base Redundancy Analysis (dbRDA), correlating **c** physicochemical properties (data set is described in Supplementary Table 1A) and **d** metabolite concentrations (data set is described in Supplementary Table 1B) with bacterial communities in bulk soil (upper panel) and rhizosphere soil (lower panel)

**Fig. 5:**
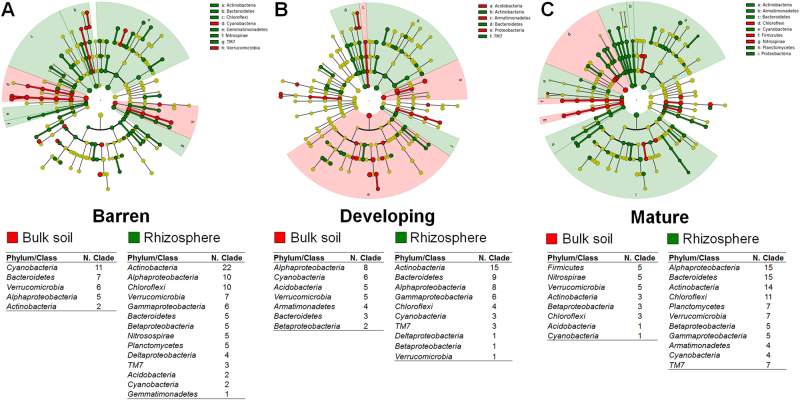
Discriminant taxa significantly retrieved by LEfSe analysis for bulk soil and rhizosphere bacterial communities at each developmental stage. The cladogram reports the taxonomic representation of statistically consistent differences between rhizosphere and bulk soil bacterial communities in **a** ‘barren’, **b** ‘developing’, and **c** ‘mature’ soils. Below each cladogram, discriminant clades for bulk soil (in red shades) and rhizosphere (in green shades) are reported. The tables underneath each cladogram report phyla/classes that statistically significantly discriminate bulk vs rhizosphere soil. Further details at higher taxonomical level are reported in the Supplementary Table 6

The rhizosphere of *S. oppositifolia* and the bulk soil were dominated by *Proteobacteria*, *Actinobacteria*, *Chloroflexi, Bacteroidetes, Acidobacteria*, and *Cyanobacteria. Proteobacteria* always dominated in both fractions, whereas *Acidobacteria* and *Chloroflexi* increased and *Bacteroidetes* and *Cyanobacteria* decreased along the chronosequence (Figs. 4a, b; Supplementary Table 5). Similar bacterial taxa distribution patterns have been observed in soil [6, 11] and the rhizosphere of *Poa alpina* [14] in moraines with increasing deglaciation time, suggesting that these taxa play relevant functional roles across soil development gradients. In all the stages, the LEfSe detected differential clades in fractions, which consistently explained the statistically significant differences between the bulk soil and rhizosphere bacterial communities (Fig. 5 and Supplementary Table 6). The number of discriminant clades decreased in bulk soil passing from ‘barren’ to ‘mature’ soils (14, 11, and 5), whereas this number increased in the rhizosphere (22, 26, and 31). In the ‘barren’ soils, LEfSe indicated *Cyanobacteria* (11), *Bacteroidetes* (7) *Verrucomicrobia* (6), and *Alphaproteobacteria* (5) among the most differentially abundant bacterial taxa in the bulk soil and *Actinobacteria* (22), *Alphaproteobacteria* (10), *Chloroflexi* (10), and *Verrucomicrobia* (7) in the rhizosphere (Fig. 5a and Supplementary Table 6). In the ‘developing’ soils, *Acidobacteria* (5) and *Armatimonadetes* (4) for the bulk soil and *Actinobacteria* (15), *Bacteroidetes* (9), and *Gammaproteobacteria* (6) for the rhizosphere, also appeared as main discriminant clades. In the ‘mature’ soils, *Firmicutes* (5), *Nitrospirae* (5), and *Verrucomicrobia* (5) were characteristic of the bulk soil, whereas *Bacteroidetes* (15), *Alphaproteobacteria* (15), *Actinobacteria* (14), and *Chloroflexi* (11) characterized the rhizosphere. From these results, we infer that bacterial groups associated with soil fertility, such as *Actinomycetales* and *Alphaproteobacteria* (*Rhizobiales* and *Sphingomonadales*, Compant et al. 2010), were increasingly enriched in the rhizosphere along the soil developmental gradient, similarly to observations in time-independent soil developmental and plant enrichment spots in the same glacier moraine [38].

### Environmental parameters related to pedogenesis shape the bacterial communities in rhizosphere and bulk soils

The concentration of key nutrients and physicochemical parameters related to soil fertility changed from ‘barren’ to ‘mature’ soils along the Midtre Lovénbreen chronosequence (Supplementary Table 1A). TOC, NTK, available phosphorous, and the TOC/NKT ratio, all influencing soil microbial communities (Knelman et al. 2014; [39, 40]), increased along the chronosequence (Supplementary Table 1A; [4]). We measured a higher relative concentration of labile carbon, indicated by a higher DOC/TOC ratio, in ‘barren’ than in ‘mature’ soils, suggesting a progressive increase of recalcitrant carbon sources in ‘mature’ soils (Supplementary Table 1A), as typically occurs during the pedogenesis process. The exometabolome measures metabolites that are promptly available for microbial metabolism and that are a function of the ‘instantaneous’ metabolic state of the microbial communities. Although the concentrations of organic acids, sugar alcohols and amino acids in the DOM (Supplementary Table 1B) was substantially stable along the chronosequence, an apparent increasing trend was observed in sugar concentration suggesting increasing organic matter deposition by the vegetation cover in the ‘mature’ soils.

The changes in bacterial community composition are explained by a significant correlation with physicochemical soil properties (Fig. 4c) and metabolite concentrations (Fig. 4d). Bacterial diversity in the bulk soil significantly correlated with P, TOC/NKT, Ca, K_exc_ and total K, Mg, Na (DistLM, AICc = 118.71, *R* ^2^ = 0.91), and, among metabolites, with sugars, amino acids, and organic acids (DistLM, AICc = 141.16, *R* ^2^ = 0.51). In another chronosequence, the belowground bacterial community correlated significantly with the amino-acid distribution [36]. Bacterial diversity in the rhizosphere significantly correlated with amino acids and sugars (DistLM, AICc = 129.15, *R* ^2^ = 0.40), and with CEC and K_exc_ (DistLM, AICc = 125.87, *R* ^2^ = 0.49), which have been linked to the water holding capacity and nutrient availability in cold desert soils [7, 17]. The highest K concentration in the ‘barren’ soils across the chronosequence was associated with rock weathering, which is initially a chemicophysical process that is enhanced by plant roots and microorganism-mediated mineral dissolution [17, 38, 41].

The prevalence of the main bacterial taxonomic groups is reflected in the physicochemical changes occurring along the chronosequence. The DOC/TOC ratio strongly decreased across the Midtre Lovénbreen chronosequence (Supplementary Table 1A), regulating the differential distribution of copiotrophs and oligotrophs such as *Acidobacteria*, a phylum including species capable of degrading plant-derived recalcitrant compounds [6, 8]. The distribution pattern of *Acidobacteria* also followed the shift in soil pH across the chronosequence. *Bacteroidetes* were more abundant in the rhizosphere of ‘barren’ soils where we measured a higher DOC/TOC ratio (Supplementary Table 1A), indicating a high relative availability of soluble organic carbon. A positive relationship between the abundance of *Bacteroidetes* and the availability of labile organic carbon has been previously demonstrated [42]. The *Cyanobacteria* distribution pattern in the rhizosphere of *S. oppositifolia* was coherent with the capacity of these bacteria to colonize recently deglaciated soils [9, 12, 38], where they contribute to enhance soil development through a soil-age-independent mechanism [17].

### OTU bacterial networks in the rhizosphere and bulk soils are diverse and subjected to the influence of soil developmental stage

**Fig. 6:**
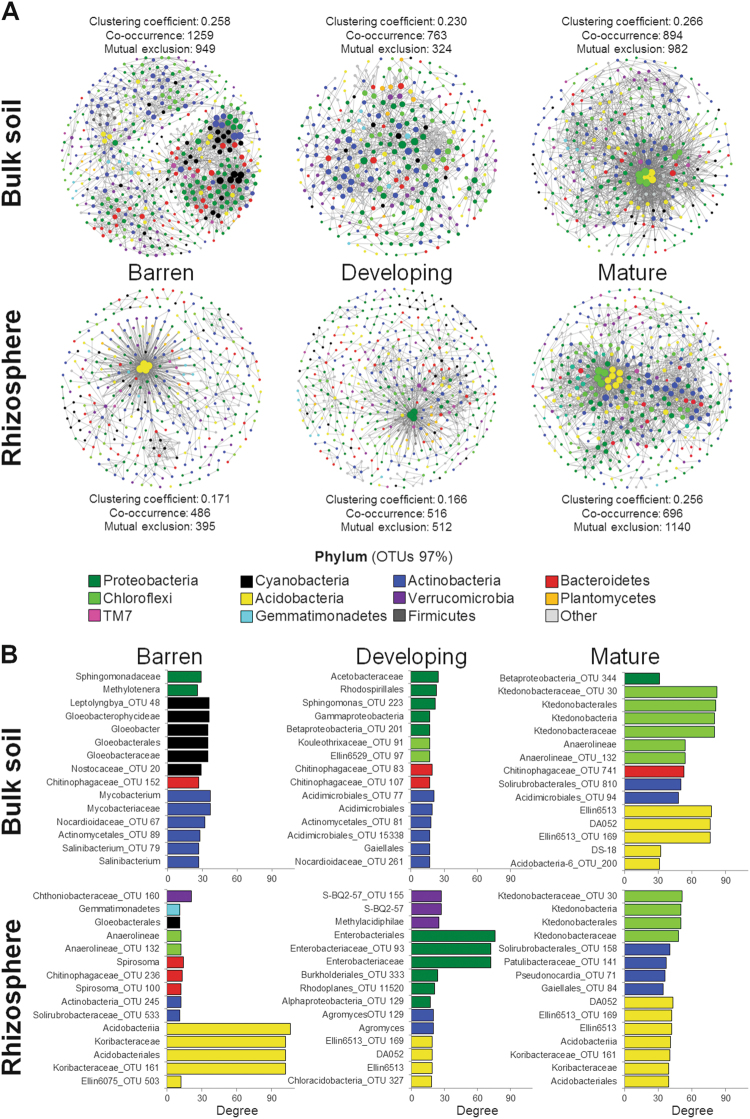
Significant co-occurrence and mutual exclusion network analysis. **a** Interaction among OTUs in the ‘barren’ (*n* = 9), ‘developing’ (*n* = 6), and ‘mature’ bulk (*n* = 6) soils and rhizospheres. For each developmental stage, the nodes correspond to the present OTUs colored according to phylum affiliation. The size of the nodes is proportional to their degree of connection (the number of edges associated to the node). **b** Bar charts indicate the most connected nodes in each network with the relative taxonomic affiliation

Bacterial OTU co-occurrence networks showed marked differences across the soil developmental stages (Fig. 6a; Supplementary Figure 4 and 5 and Supplementary Table 7). Although the number of nodes increased in the ‘mature’ bulk soil (474, 332, and 1877 nodes in ‘barren’, ‘developing’, and ‘mature’ soils, respectively), this number was less variable in the rhizosphere (360, 443, and 378 nodes, respectively). Despite the observed variability in the node number in bulk soils, network clustering coefficient did not vary across the chronosequence (Fig. 6a, Supplementary Figure 4B). The number of interactions (edges) dropped from 2208 to 1087 from the ‘barren’ to the ‘developing’ stages and increased to 1876 at the ‘mature’ stage, indicating a high turnover of OTUs serving as connections, without effects on parameters of the overall network topology (Supplementary Figure 5). This suggests an ecological vicariance of OTUs that serve as connection nodes (Supplementary Figure 4) [43]. Conversely, in the rhizosphere the connections and the clustering coefficient both increased in the ‘mature’ soils, from 880 up to 1836 and from 0.171 to 0.256, respectively (Fig. 6a; Supplementary Figure 4B).

The analysis of the topological coefficient revealed similar trends in the bulk soil and rhizosphere networks (Supplementary Figure 5). For all three stages, we recorded a higher topological coefficient for the rhizosphere than the bulk soil, indicating that OTUs in the rhizosphere have a higher tendency to share neighbors. The magnitude of this difference decreased with soil development. An opposite trend was recorded for the node degree of distribution (Supplementary Figure 5). At the ‘barren’ stage, bulk soil had a higher level of node degree distribution than rhizosphere, but the reverse trend was observed at the ‘mature’ stage. In the bulk soil, betweenness centrality of the majority of nodes was low at all three stages of soil development, with only few low-number-of-neighbors nodes with high values of betweenness centrality in the ‘barren’ and the ‘developing’ soils. The rhizosphere presented a relatively high-number of low-number-of-neighbors nodes with medium to high betweenness centrality in the ‘barren’ and the ‘developing’ soils, whereas in the ‘mature’ soils most of the nodes were high-number-of-neighbors with low values of betweenness centrality (Supplementary Figure 5). The observed betweenness centrality distribution of the network nodes indicates that key networking roles are taken by several nodes in the ‘barren’ and ‘developing’ soil stages, especially in the rhizosphere, and that the bacterial community underwent re-arrangements that confer crucial roles to several OTUs in defining the overall network structure. At the ‘mature’ soil stage, a large proportion of nodes defines the network topology in both the fractions, suggesting that a higher number of OTUs shape the structure of the bacterial community interactions.

The taxonomical distributions of node degrees at the ‘barren’ soil stage indicated that the rhizosphere contained highly connected nodes belonging to *Acidobacteria* (Fig. 6b). This suggests an important ecological role of such a phylum in the assembly of the bacterial community at the early phases of soil development. In the ‘developing’ soil stage, *Proteobacteria* (*Enterobacterales*) drove rhizosphere community connectivity, whereas in the ‘mature’ soil *Choloroflexi*, *Actinobacteria*, and *Acidobacteria* became the main players in shaping the topology of the bacterial network. Bulk soils were characterized by an even distribution of node connection degrees, mainly represented by *Cyanobacteria* and *Actinobacteria* in the ‘barren’ soils and *Proteobacteria* and *Actinobacteria* in the ‘developing’ soils. In the ‘mature’ soils, the taxonomical distributions of node degrees in the bulk soil converged toward one similar to that in the rhizosphere, but with a high degree of connections of *Chloroflexi* and *Acidobacteria* phyla.

Our bacterial network analysis indicates that even though plant-root-related processes (such as rhizodeposition) impact the overall network topology of the rhizosphere across the chronosequence, the selective pressure imposed by the heterogeneous and evolving soil conditions drives the phylogenetic assembly of the bacterial communities in both soil fractions. The rhizospheres of the plant *Avena fatua* grown in mature soils have more complex bacterial networks compared with bulk soil, due to the higher organization of the bacterial community as reflected in increased interactions and niche sharing [44]. In contrast, during early soil development stages, our results indicate that bulk soils have a more complex level of bacterial network organization and connectivity than rhizosphere soils. Previous studies in recently deglaciated substrates corroborate that bacterial community composition is more strongly influenced by the harsh conditions of the barren substrates than by the plant effect [14, 45, 46]. Our results show an increase in the number of edges occurring across the different soil developmental stages, suggesting that the complexity of the rhizosphere community increases with soil maturity. Manipulative experiments have shown that soil composition strongly drives the microbial communities in the rhizosphere and their stability, even though plant roots impose strong selection pressure [47, 48]. Our data support the notion that during soil formation, the plant-root-imposed selection pressure is strong enough to shape the rhizosphere-specific bacterial community structure and, at the same time, the developmental stage of the soil tunes the networking properties of the bacterial community in the rhizosphere, ultimately shaping its assembly. The observed effect of the soil developmental stage on a plant’s rhizosphere assembly contributes to our understanding of the plant-supportive soil formation processes in barren desert ecosystems and, by a reverse analogy, the loss of such properties occurring during desertification.

## Electronic supplementary material

Supplementary Information

Supplementary Information Table S6

Supplementary Information Table S7

## References

[1] Makhalanyane TP, Valverde A, Velázquez D, Gunnigle E, Van Goethem MW, Quesada A (2015). Ecology and biogeochemistry of cyanobacteria in soils, permafrost, aquatic and cryptic polar habitats. Biodivers Conserv.

[2] Philippot L, Raaijmakers JM, Lemanceau P, van der Putten WH (2013). Going back to the roots: the microbial ecology of the rhizosphere. Nat Rev Microbiol.

[3] Berg G, Smalla K (2009). Plant species and soil type cooperatively shape the structure and function of microbial communities in the rhizosphere. FEMS Microbiol Ecol.

[4] Hodkinson ID, Coulson SJ, Webb NR (2003). Community assembly along proglacial chronosequences in the high Arctic: vegetation and soil development in north-west Svalbard. J Ecol.

[5] Walker LR, Wardle DA, Bardgett RD, Clarkson BD (2010). The use of chronosequences in studies of ecological succession and soil development. J Ecol.

[6] Nemergut DR, Anderson SP, Cleveland CC, Martin AP, Miller AE, Seimon A (2007). Microbial community succession in an unvegetated, recently deglaciated soil. Microb Ecol.

[7] Noll M, Wellinger M (2008). Changes of the soil ecosystem along a receding glacier: testing the correlation between environmental factors and bacterial community structure. Soil Biol Biochem.

[8] Rime T, Hartmann M, Brunner I, Widmer F, Zeyer J, Frey B (2015). Vertical distribution of the soil microbiota along a successional gradient in a glacier forefield. Mol Ecol.

[9] Schmidt S, Reed SC, Nemergut DR, Stuart Grandy A, Cleveland CC, Weintraub MN (2008). The earliest stages of ecosystem succession in high-elevation (5000 metres above sea level), recently deglaciated soils. Proc R Soc B.

[10] Schütte UME, Abdo Z, Bent SJ, Williams CJ, Schneider GM, Solheim B (2009). Bacterial succession in a glacier foreland of the High Arctic. ISME J.

[11] Schütte UME, Abdo Z, Foster J, Ravel J, Bunge J, Solheim B (2010). Bacterial diversity in a glacier foreland of the high Arctic. Mol Ecol.

[12] Zumsteg A, Luster J, Göransson H, Smittenberg RH, Brunner I, Bernasconi SM (2012). Bacterial, archaeal and fungal succession in the forefield of a receding glacier. Microb Ecol.

[13] Brown SP, Jumpponen A (2015). Phylogenetic diversity analyses reveal disparity between fungal and bacterial communities during microbial primary succession. Soil Biol Biochem.

[14] Tscherko D, Hammesfahr U, Marx MC, Kandeler E (2004). Shifts in rhizosphere microbial communities and enzyme activity of Poa alpina across an alpine chronosequence. Soil Biol Biochem.

[15] Deiglmayr K, Philippot L, Tscherko D, Kandeler E (2006). Microbial succession of nitrate-reducing bacteria in the rhizosphere of Poa alpina across a glacier foreland in the Central Alps. Environ Microbiol.

[16] Vanderpuye A, Arve E, Lennard N (2002). Plant communities along environmental gradients of high-arctic mires in Sassendalen, Svalbard. J Veg Sci.

[17] Borin S, Ventura S, Tambone F, Mapelli F, Schubotz F, Brusetti L (2010). Rock weathering creates oases of life in a High Arctic desert. Environ Microbiol.

[18] Scaglia B, Adani F (2009). Biodegradability of soil water soluble organic carbon extracted from seven different soils. J Environ Sci (China).

[19] Scaglia B, Pognani M, Adani F (2015). Evaluation of hormone-like activity of the dissolved organic matter fraction (DOM) of compost and digestate. Sci Total Environ.

[20] Anderson MJ, Willis TJ (2003). Canonical analysis of principal coordinates: a useful method of constrained ordination for ecology. Ecology.

[21] Cardinale M, Brusetti L, Quatrini P, Borin S, Puglia AM, Rizzi A (2004). Comparison of different primer sets for use in automated ribosomal intergenic spacer analysis of complex bacterial communities. Appl Environ Microbiol.

[22] Lane DJ. 16S/23S rRNA sequencing. In E. Stackebrandt and M. Goodfellow (ed.), Nucleic acid techniques in bacterial systematics. John Wiley & Sons, New York. 1991;115–175.

[23] Dini-Andreote F, Stegen JC, van Elsas JD, Salles JF (2015). Disentangling mechanisms that mediate the balance between stochastic and deterministic processes in microbial succession. Proc Natl Acad Sci USA.

[24] Anderson MMJ, Gorley RNRN, Clarke KR. PERMANOVA+ for PRIMER: guide to software and statistical methods. Plymouth, UK; 2008.

[25] Segata N, Izard J, Waldron L, Gevers D, Miropolsky L, Garrett WS (2011). Metagenomic biomarker discovery and explanation. Genome Biol.

[26] Faust K, Sathirapongsasuti JF, Izard J, Segata N, Gevers D, Raes J (2012). Microbial co-occurrence relationships in the human microbiome ouzounis CA (ed). PLoS Comput Biol.

[27] Barberán A, Bates ST, Casamayor EO, Fierer N (2012). Using network analysis to explore co-occurrence patterns in soil microbial communities. ISME J.

[28] Bastian M, Heymann S, Jacomy M. Gephi: an open source software for exploring and manipulating networks. Third Int AAAI Conf Weblogs Soc Media. 2009; 361-2.

[29] Rosenvald K, Kuznetsova T, Lõhmus K, Ostonen I, Truu M, Truu J (2011). Dynamics of rhizosphere processes and soil formation in a chronosequence of silver birch stands on reclaimed oil shale post-mining areas. Ecol Eng.

[30] Kumar M, Brader G, Sessitsch A, Mäki A, van Elsas JD, Nissinen R (2017). Plants assemble species specific bacterial communities from common core taxa in three arcto-alpine climate zones. Front Microbiol.

[31] Kumar M, Männistö MK, van Elsas JD, Nissinen RM (2016). Plants impact structure and function of bacterial communities in Arctic soils. Plant Soil.

[32] Coleman-Derr D, Desgarennes D, Fonseca-Garcia C, Gross S, Clingenpeel S, Woyke T (2016). Plant compartment and biogeography affect microbiome composition in cultivated and native Agave species. New Phytol.

[33] Miniaci C, Bunge M, Duc L, Edwards I, Bürgmann H, Zeyer J (2007). Effects of pioneering plants on microbial structures and functions in a glacier forefield. Biol Fertil Soils.

[34] Yergeau E, Newsham KK, Pearce DA, Kowalchuk GA (2007). Patterns of bacterial diversity across a range of Antarctic terrestrial habitats. Environ Microbiol.

[35] Chandler DP, Fredrickson JK, Brockman FJ (1997). Effect of PCR template concentration on the composition and distribution of total community 16S rDNA clone libraries. Mol Ecol.

[36] Moon J, Ma L, Xia K, Williams MA (2016). Plant – microbial and mineral contributions to amino acid and protein organic matter accumulation during 4000 years of pedogenesis. Soil Biol Biochem.

[37] Dombrowski N, Schlaeppi K, Agler MT, Hacquard S, Kemen E, Garrido-Oter R (2017). Root microbiota dynamics of perennial Arabis alpina are dependent on soil residence time but independent of flowering time. ISME J.

[38] Mapelli F, Marasco R, Rizzi A, Baldi F, Ventura S, Daffonchio D (2011). Bacterial communities involved in soil formation and plant establishment triggered by pyrite bioweathering on Arctic moraines. Microb Ecol.

[39] Kuramae EE, Yergeau E, Wong LC, Pijl AS, Veen JA, Kowalchuk GA (2012). Soil characteristics more strongly influence soil bacterial communities than land-use type. FEMS Microbiol Ecol.

[40] Ramirez KS, Lauber CL, Knight R, Bradford MA, Fierer N (2010). Consistent effects of nitrogen fertilization on soil bacterial communities in contrasting systems. Ecology.

[41] Mapelli F, Marasco R, Balloi A, Rolli E, Cappitelli F, Daffonchio D (2012). Mineral–microbe interactions: biotechnological potential of bioweathering. J Biotechnol.

[42] Fierer N, Bradford MA, Jackson RB (2007). Toward an ecological classification of soil bacteria. Ecology.

[43] Marske KA, Rahbek C, Nogués-Bravo D (2013). Phylogeography: spanning the ecology-evolution continuum. Ecography (Cop).

[44] Shi S, Nuccio EE, Shi ZJ, He Z, Zhou J, Firestone MK (2016). The interconnected rhizosphere: high network complexity dominates rhizosphere assemblages. Ecol Lett.

[45] Edwards IP, Bürgmann H, Miniaci C, Zeyer J (2006). Variation in microbial community composition and culturability in the rhizosphere of Leucanthemopsis alpina (L.) heywood and adjacent bare soil along an alpine chronosequence. Microb Ecol.

[46] Tscherko D, Hammesfahr U, Zeltner G, Kandeler E, Böcker R (2005). Plant succession and rhizosphere microbial communities in a recently deglaciated alpine terrain. Basic Appl Ecol.

[47] Tkacz A, Cheema J, Chandra G, Grant A, Poole PS (2015). Stability and succession of the rhizosphere microbiota depends upon plant type and soil composition. ISME J.

[48] Yan Y, Kuramae EE, de Hollander M, Klinkhamer PGL, van Veen JA (2017). Functional traits dominate the diversity-related selection of bacterial communities in the rhizosphere. ISME J.

