# Supplementary material for: The stage of soil development modulates rhizosphere effect along a High Arctic desert chronosequence
Source: ISME J. 2018 Jan 15;12(5):1188–98. doi: 10.1038/s41396-017-0026-4 (PMC5931989; doi:10.1038/s41396-017-0026-4)
Supplement: Supplementary file 1 — Supplementary Information [file 41396_2017_26_MOESM1_ESM.docx]

**SUPPLEMENTARY INFORMATION to manuscript**

**The stage of soil development modulates rhizosphere effect along a High Arctic desert chronosequence**

Francesca Mapelli^1#^, Ramona Marasco^2#^, Marco Fusi^2#^, Barbara Scaglia^3^, George Tsiamis^4^, Eleonora Rolli^1^, Stilianos Fodelianakis^2^, Kostas Bourtzis^4^, Stefano Ventura^5^, Fulvia Tambone^3^, Fabrizio Adani^3^, Sara Borin^1^* and Daniele Daffonchio^2^*

*^1^ Department of Food, Environmental and Nutritional Sciences, University of Milan, Milan 20133, Italy*

*^2^ King Abdullah University of Science and Technology (KAUST), Biological and Environmental Sciences and Engineering Division (BESE), Thuwal 23955-6900, Saudi Arabia*

*^3^ Department of Agricultural and Environmental Sciences - Production, Landscape, Agroenergy, University of Milan, Milan 20133, Italy*

*^4^ Department of Environmental and Natural Resources Management, University of Patras, Agrinio 30100, Greece*

*^5^ Institute of Ecosystem Study, CNR, Sesto Fiorentino 50019, Italy*

* **Corresponding authors:** Daniele Daffonchio, King Abdullah University of Science and Technology (KAUST), BESE Division, Thuwal, 23955-6900, Kingdom of Saudi Arabia. Phone: +966 (2) 8082884; E-mail: daniele.daffonchio@kaust.edu.sa; Sara Borin, Università degli Studi di Milano DeFENS, Via Celoria 2, 20133 Milan, Italy. Phone: +390250319118; Fax: +390250319238; E-mail: sara.borin@unimi.it.

**#:** These authors contributed equally

**Running title**

*Soil development modulates rhizosphere effect*

**SUPPLEMENTARY INFORMATION LIST**

**SUPPLEMENTARY METHODS**

**Supplementary Method S1**

**Supplementary Method S2**

**Supplementary Method S3**

**Supplementary Method S4**

**Supplementary Method S5**

**Supplementary Method S6**

**SUPPLEMENTARY TABLES**

**Supplementary Table S1**

**Supplementary Table S2**

**Supplementary Table S3**

**Supplementary Table S4**

**Supplementary Table S5**

**Supplementary Table S6 (attached excel file)**

**Supplementary Table S7 (attached excel file)**

**SUPPLEMENTARY FIGURES**

**Supplementary Figure S1**

**Supplementary Figure S2**

**Supplementary Figure S3**

**Supplementary Figure S4**

**Supplementary Figure S5**

**SUPPLEMENTARY REFERENCES**

**SUPPLEMENTARY METHODS**

**Supplementary Method S1**

Soil parameters were determined as follows: Soil surrounding root pH was determined in aqueous solution using a 1:2.5 sample/water ratio. Total nitrogen was determined by the Kjeldahl method. Available-P was determined by the Olsen method. Organic carbon was determined by wet oxidation. For determining cation exchange capacity (CEC), samples were saturated with BaCl_2_-Triethanolamine solution (pH 8.1) and exchangeable cations (Ca exch, Mg exch, Na exch, Ca exch) were determined by Inductively Coupled Plasma (ICP-MAS VARIAN, Liberty AX, Walnut Creek, CA). Total Ca, Mg, K, Na, Mn and P were determined by samples digestion with HNO_3_ (16 mol l^-1^) in a microwave furnace (CEM Mars 5, Matthevs, North Caroline), and successive detection by Inductively Coupled Plasma. All the above methods were described by Borin and coworkers (Borin *et al.*, 2010). Soil respirations were measured by trapping with alkali the CO_2_ produced during soil incubation at 20°C in the laboratory for 21 days (Scaglia *et al.*, 2015).

Dissolved organic matter (DOM) was extracted with deionized water from ten grams of each soil. The extraction was done by using soil/water ratio of 1:2 weight/volume for 30 min, at room temperature under agitation (130 times/min) in a Dubnoff bath. After the extraction, samples were centrifuged at 6500 r/min for 15 min. Supernatants were filtered with a 0.45-µm Millipore membrane (Advantec MFS, Pleasanton, CA) and dissolved organic carbon (DOC) was quantified by organic carbon determination as reported previously.

Analysis of DOM chemical composition was performed by using GC/MS as reported previously (Scaglia *et al.*, 2015). Briefly, DOM fractions were evaporated to dryness under vacuum conditions. Ten microliters of methoxyamine hydrochloride (Sigma Aldrich, 33045) in pyridine (Sigma Aldrich, 360570) at a concentration of 40 mg ml^−1^ was added to the samples and the mixtures were shaken at 30°C for 90 min. Subsequently, 90 μl of N-methyl-Ntrimethylsilyltrifluoroacetamide with 1% trimethylchlorosilane (Sigma Aldrich, 3-3148) was added to samples and incubated at 37°C for 30 min. DOM chemical composition was determined using an Agilent 5975C Series GC/MSD. Molecules were separated using a capillary column ZB-Semivolatiles 30 m × 250 μm × 0.25 μm (ID). The carrier gas was helium at a flow rate of 1.1 ml min^−1^. One microliter of sample was injected using CTC PAL into the GC injection port at 250°C in splitless mode. The temperature program was set at 60°C for 1 min, raised to 325°C at a rate of 10°C/min and the final temperature was maintained for 10 min. The transfer line to the mass spectrometer was maintained at 290°C. The mass spectra were obtained by electronic impact at 70 eV, and data were collected at an m/z range of 50-660. Compounds were tentatively identified by comparing their mass spectra with those contained in the Agilent Fiehn GC/MS Metabolomics RTL Library (Agilent Technologies, Inc. 2008) using AMDIS software (Automated Mass Spectral Deconvolution and Identification System software, NIST08).

**Supplementary Method S2**

The automated ribosomal intergenic spacer analysis (ARISA) fingerprinting was performed accordingly to the protocol described in Cardinale and coworkers (Cardinale *et al.*, 2004) using the primer set ITSF, 5’-GTCGTAACAAGGTAGGCCGTA-3’ and ITSReub, 5’-GCCAAGGCATCCACC-3’. The PCR reaction mixture contained 1X PCR buffer, 1.5 U of Taq DNA polymerase (Invitrogen), 0.2 mM (each) dNTPs, and 0.25 µM (each) primer in a final volume of 25 µL. The mixture was held at 94°C for 3 min, followed by 30 cycles of 94°C for 45 s, 55°C for 1 min, 72°C for 2 min, and a final extension at 72°C for 7 min. The ARISA peak patterns were analysed using GeneScan 3.1 software and a quantitative matrix reporting the relative abundance of each peak was analysed. To account for variability in size associated with standards, ARISA fingerprints were binned ±1 bp from150 to 300 bp, ±3 bp from 300 to 500 bp and ±10 bp > 500 bp (Mapelli *et al.*, 2013).

For PhyloChip analysis, a reaction mixture (20 μl) containing the PCR buffer supplied by the polymerase manufacturer, 1.5 mM MgCl_2_, 0.25 mM of each deoxynucleoside triphosphate, 0.3 mM of the 27F and 1492R primers, and 1 U Taq polymerase (Takara Mirus Bio Inc., WI) was prepared. PCR reactions were performed using a PTC-200 thermocycler (MJ Research Inc., USA) with a denaturation step of 10 min at 94°C, followed by 35 cycles for library construction and 30 cycles for the PhyloChip analysis: 1 min denaturation at 94°C, 1 min primer annealing at 52°C for the library construction; for the PhyloChip analysis, eight annealing temperatures between 48°C and 58°C were used, and finally 90 s extension at 72°C. The PCR was completed by a final extension at 72°C for 10 min. The size of the PCR products was determined by agarose gel electrophoresis using appropriate size markers. The DNA mixture of the soil amplicons and internal standard was fragmented to 50-200 bp with DNase I (0.02 U/μg DNA; Invitrogen, USA) in 10x DNase I Buffer (USB, USA), according to the Affymetrix protocol (Affymetrix, USA). The fragmented material was then labelled with biotin using GeneChip DNA labelling reagent (Affymetrix, USA) and terminal deoxynucleotide transferase (Promega, USA) according to the manufacturer’s instructions. Before hybridization to the PhyloChip, the labelled DNA was denatured at 99°C for 5 min. The hybridization reaction was carried out at 48°C for 16 h with shaking at 60 rpm. All washing and scanning was performed with a GeneArray Scanner (Affymetrix, USA) as previously described^6^. Probe pairs, from three independent experiments that met the following two criteria were scored as positive: (1) the fluorescence intensity of the perfectly matched probe was at least 1.3 times greater than the intensity of the mismatched control; and (2) the difference in intensity, perfectly matched minus mismatched control, was at least 130 times greater than the squared noise value (>130*N^2^*). A positive fraction of 0.95, which is hybridization of more than 95% of taxon-specific probes, was our threshold for detection and positive identification of a specific taxon within a sample (Tsiamis *et al.*, 2008; Brodie *et al.*, 2006).

To evaluate the significant differences among the two fraction (bulk and rhizosphere) Bray-Curtis dissimilarity matrices were calculated from the relative abundance of bacteria distribution tables, obtained by the PhyloChip and the ARISA analyses, using the PERMANOVA analysis. A Principal Coordinates Analysis (PCoA) was also performed to assess the phylogenetic β-diversity. All the statistical analyses were performed by PRIMER v. 6.1, PERMANOVA+ for PRIMER routines (Anderson *et al.*, 2008).

**Supplementary Method S3**

Illumina tag screening of the V4-V5 hypervariable regions of the 16S rRNA gene was applied on rhizospheric and bulk soil DNA and performed at Macrogen S.A. using primers 341f and 785r (Klindworth *et al.*, 2013). The obtained sequences were analysed using a combination of the UPARSE v8 (Edgar, 2013) and the QIIME v1.8 (Caporaso *et al.*, 2010) softwares. Briefly, raw forward and reverse reads for each sample were assembled into paired-end reads considering a minimum overlapping of 50 nucleotides and a maximum of one mismatch within the region using the fastq-join algorithm (https://code.google.com/p/ea-utils/wiki/FastqJoin). The paired reads were then quality filtered, the primer sequences were removed and the individual sample files were merged in a single fasta file. This file was imported in UPARSE where operational taxonomic units (OTUs) of 97% sequence similarity were formed and chimeras were removed using both de-novo and reference-based detection. For reference chimera detection, the “Gold” database containing the chimera-checked reference database in the Broad Microbiome Utilities (http://microbiomeutil.sourceforge.net/) was used. Taxonomy was assigned to the representative sequences of the OTUs in QIIME using UClust (Edgar, 2010) and searching against the latest version of the Greengenes database (McDonald *et al.*, 2012). Rarefactions were assessed and all samples had a coverage of more than 99% (see Supplementary Figure S5). Finally, an OTU table (i.e., a sample x OTU count matrix with a tab containing the taxonomic affiliation of each OTU) was created. The OTU table and the phylogenetic tree were calculated with FastTree2 (Price *et al.*, 2010) using default parameters and the PyNast-aligned representative sequences as an input. The OTU table and the phylogenetic tree were used as inputs for the subsequent analyses of alpha- and beta-diversity (UniFrac weighted and unweighted).

**Supplementary Method S4**

To set up the developmental stage groups along the chronosequence we used the Canonical Analysis of Principal coordinates (Anderson, 2002) on the chemical characteristics of the soils. The physico-chemical matrix (see Supplementary Table S1A) containing the data from each of the seven sampled sites was firstly normalized and Spearman’s correlation was performed in order to eliminate covariate variables. The normalized matrix was used to create a resemblance matrix using the Euclidean distance. We used such matrix to perform the Canonical Analysis of Principal coordinates and to perform the Similarity Percentage Analysis (SIMPER) in order to estimate the more important chemical variables contributing to define the developmental stages (see Supplementary Table S1C-D). The choice of this analysis relies on the fact that it takes into account the correlation structure among the variables following the more traditional multivariate discriminant (or canonical) analysis, but it does this on the principal coordinates from the distance matrix. In the Supplementary Figure S2, the cross validation table, statistic delta and the figure output underlying the three different groups. The cross validation evidences that Canonical Analysis of Principal coordinates can allocate correctly all samples in the relative groups.

To test the bacterial compositional differences across the chronosequence, we performed a Permutational multivariate analysis of the variance on Bray-Curtis, unweighted and weighted UniFrac distance matrices (Lozupone *et al.*, 2011) using the Illumina 16S rRNA gene-base dataset, considering as categorical variable the Soil Development State (three levels: ‘Barren’, ‘Developing’ and ‘Mature’) and Soil Fraction (two levels: ‘Bulk soil’ and ‘Rhizosphere’).

Prior to run the Permanova we test the homogeneity of the dispersions of the categorical variables using PERMDISP for the three distances matrices. For Fraction (unweighted UniFrac F_1,39_=5.80, p=0.06; weighted UniFrac, F_1,39_=4.48; p=0.06; Bray-Curtis: F_1,39_=4.19; p=0.13) and Soil Development State (uweighted UniFrac F_2,38_=2,9, p=0.11; weighted UniFrac, F_2,38_=3.6; p=0.07; Bray-Curtis: F_2,38_=1.98; p=0.19) we detected no statistical significance of the dispersion.

Linear Discriminant Analysis (Wilcoxon p-value: 0.05, LDA>2) was used to assess discriminant taxa characterizing the bulk soil and the rhizosphere at each developmental stage (Segata *et al.*, 2011).

**Supplementary Method S5**

Distance-based multivariate analysis for a linear model (DistLM) was carried out to determine the environmental variables explaining the community variance among the samples (Anderson *et al.*, 2008). The corrected Akaike information criterion (AICc) was used to assess the significance of the predictor variables (Konishi and Kitagawa, 2008). Multi-collinearity among the environmental variables was checked using the non-parametric Spearman correlation and the Draftsman’s plots. Variables having a correlation coefficient higher than 0.85, were removed from subsequent analyses.

**Supplementary Method S6**

Using the routine CoNet in Cytoscape 3.2.1 (Faust *et al.*, 2012) we built a co-occurrence network to find OTUs significantly co-existing or mutually excluded across the chronosequence. We used as input data the OTU table after removing the rare OTUs (less than 0.1% of sequences per sample). To build the network, after filtering out the less frequent than 0.05, we combined an ensemble of the Pearson and Spearman correlation coefficients, and the Bray-Curtis (BC) and Kullback-Leibler (KLD) dissimilarity indices. To compute the statistical significance of the co-occurrence/mutual exclusion we first computed edge-specific permutation and bootstrap score distributions with 1,000 iterations; we re-normalized the data in each permutation, providing a null distribution that captures the similarity introduced by compositionality alone. We then computed the p-value as above by z-scoring the permuted null and bootstrap confidence interval using pooled variance as explained by Barberán and coworkers (Barberán *et al.*, 2012). The clustering coefficients (that is a measure that identify a modular organization of networks), the Degree connectivity distribution (number of edges linked to the node), betweeness centrality (that reflects the amount of control that this node exerts over the interactions of other nodes in the network) and the topological coefficient (the tendency of the nodes in the network to have shared neighbors) as well as degree of connection of each node, number of positive (co-occurrence) and negative (mutual exclusion) edge were calculated among the most important statistical descriptor of the network (Doncheva *et al.*, 2012). These structural properties offer the potential for quick and easiest comparisons among complex datasets from different stages of soil formation in order to explore how the time of glacier retreat can determine the assembly of bacterial communities in the bulk soil and the rhizosphere. Network visualization was performed using Gephi 1.9 (Bastian *et al.*, 2009).

**SUPPLEMENTARY TABLES**

**Supplementary Table S1. (A) Physicochemical and (B) Metabolite composition of the soil samples collected from the seven Midtre Lovénbreen sites.** NTK: Total Nitrogen; TOC: Total Organic Carbon; CEC: Cation Exchange Capacity; exc: exchangeable; tot: total; DOC: Dissolved Organic Carbon. P, K, Ca, Mg, Na, Mn concentrations are expressed as mg kg^-1^ DM; NTK and TOC concentration are expressed as g kg^-1^ DM; DOC concentration is expressed as mg kg^-1^ DM. (C-E) Similarity Percentage (SIMPER) analysis on the physicochemical dataset showing the contribution of each variable to the differences between ‘barren’ and ‘developing’ (C), ‘barren’ and ‘mature’(D) and ‘developing’ and ‘mature’ (E) soil developmental stages. For each SIMPER table “Contribution (%)” refers to the contribution of the single variables, while “Cumulative (%)” refers to the cumulative difference of all the variables considered.

**(A)** **Physicochemical data of soil influenced by plant root system**

| **Physico-chemical analysis** | **Deglaciation time (years)** | | | | | | |
| --- | --- | --- | --- | --- | --- | --- | --- |
|  | **8** | **22** | **43** | **66** | **106** | **156** | **>1900** |
| pH | 8.17 | 7.69 | 8.04 | 8.08 | 8.05 | 7.72 | 6.17 |
| NTK | 0.15 ± 0.02 | 0.32 ± 0.19 | 0.43 ± 0.38 | 0.55 ± 0.12 | 0.26 ± 0.03 | 0.78 ± 0.44 | 3.1 ± 0.47 |
| TOC | 0.75 ± 0.15 | 5.22 ± 3.52 | 4.48 ± 4.43 | 9.56 ± 1.42 | 10.86 ± 2.38 | 12.5 ± 6.86 | 47.64 ± 8.32 |
| TOC/NTK | 5.3 ± 1.14 | 16.86 ± 2.86 | 6.24 ± 4.9 | 16.76 ± 4.23 | 21.62 ± 4.94 | 20.06 ± 2.47 | 16.4 ± 1.98 |
| P available | 26.55 ± 0.64 | 28.8 ± 7.02 | 34 ± 5.55 | 40.17 ± 5.42 | 40.03 ± 3.78 | 43.3 ± 7.9 | 60.57 ± 8.62 |
| CEC | 6.41 ± 0.11 | 8.66 ± 1.26 | 6.62 ± 0.7 | 10.94 ± 1.17 | 10.82 ± 2.09 | 15.53 ± 0.89 | 42.78 ± 2.31 |
| K exc | 566.05 ± 6.41 | 148.32 ± 14.02 | 172.42 ± 10.17 | 135.55 ± 4.42 | 170.68 ± 52.83 | 149.58 ± 6.11 | 141.16 ± 12.29 |
| Ca exc | 738.59 ± 8.63 | 143.28 ± 8.06 | 331.67 ± 5.79 | 177.64 ± 16.78 | 287.65 ± 14.52 | 147.95 ± 20.67 | 708.78 ± 9.16 |
| Mg exc | 827.27 ± 9.8 | 828.57 ± 12.45 | 736.14 ± 47.72 | 592.37 ± 8.44 | 488.59 ± 366.3 | 582.33 ± 52.89 | 613.72 ± 18.94 |
| Na exc | 92.07 ± 7 | 26.73 ± 3.55 | 26.38 ± 3.02 | 27.96 ± 6.42 | 36.37 ± 6.08 | 21.91 ± 4.84 | 42.49 ± 32.38 |
| Ca tot | 6384 ± 729.73 | 4155.33 ± 979.76 | 7276.33 ± 785.25 | 5735.33 ± 86.59 | 5120.67 ± 719.72 | 3895.33 ± 566.88 | 2499.67 ± 66.16 |
| K tot | 3509 ± 100.41 | 2854.33 ± 151.44 | 3518.67 ± 103.58 | 3256.33 ± 80.43 | 3075.67 ± 186.18 | 2544.67 ± 278.06 | 1650.67 ± 279.93 |
| Na tot | 232 ± 28.28 | 170 ± 28 | 158.17 ± 8.04 | 140 ± 21.79 | 136.33 ± 20.03 | 110 ± 10 | 130.07 ± 27.61 |
| Mg tot | 6149 ± 14.14 | 5864.67 ± 33.5 | 6044.33 ± 176.09 | 4650.67 ± 236.17 | 5633.33 ± 236.16 | 5036.33 ± 166.79 | 4044.33 ± 169.25 |
| Mn tot | 293 ± 12.73 | 398.33 ± 105.16 | 412 ± 14.42 | 508 ± 70.34 | 448.33 ± 34.49 | 399.67 ± 33.32 | 303 ± 7.21 |
| P tot | 796 ± 76.37 | 520.67 ± 33.23 | 822.67 ± 122.45 | 638 ± 27.22 | 711.33 ± 172.39 | 551.67 ± 27.15 | 617 ± 8.19 |
| DOC | 0.51 ± 0.06 | 0.66 ± 0.14 | 0.57 ± 0.1 | 1.37 ± 0.18 | 1.06 ± 0.22 | 1.39 ± 0.2 | 1.3 ± 0.23 |
| DOC/TOC | 56.85 ± 12.37 | 13.24 ± 8.71 | 32.33 ± 17.62 | 13.8 ± 2.57 | 8.17 ± 3.55 | 9.1 ± 5.2 | 2.83 ± 0.7 |
| CO_2_/soil | 0.63 ± 0.1 | 1.65 ± 0.22 | 3.21 ± 1.04 | 3.87 ± 1.75 | 3.86 ± 2.15 | 5.76 ± 1.16 | 8.62 ± 1.65 |
| CO_2_/C | 1271.44 ± 402.6 | 398.17 ± 214.47 | 501.76 ± 159.77 | 336.32 ± 169.05 | 335.33 ± 145.14 | 455.07 ± 157.37 | 219.27 ± 38.57 |

**(B) Metabolite concentration in soil influenced by plant root system**

| **Metabolite (% tot)** |  | **Deglaciation time (years)** | | | | | | |
| --- | --- | --- | --- | --- | --- | --- | --- | --- |
|  |  | **8** | **22** | **43** | **66** | **106** | **156** | **>1900** |
| Organic acids |  | 4.125 ± 0.358 | 7.751 ± 0.691 | 2.057 ± 0.389 | 1.23 ± 1.049 | 3.088 ± 1.173 | 1.633 ± n.d | 11.058 ± 2.234 |
| Sugar alcohols |  | 96.015 ± 0.35 | 82.922 ± 4.046 | 93.906 ± 5.427 | 92.867 ± 8.086 | 83.938 ± 5.419 | 89.815 ± n.d | 62.872 ± 7.972 |
| Amino acids |  | 0.516 ± 0.306 | 2.266 ± 0.659 | 0.391 ± 0.142 | 0.926 ± 0.416 | 0.538 ± 0.236 | 0.384 ± n.d | 0.57 ± 0.154 |
| Sugars |  | 0 ± 0 | 9.328 ± 4.673 | 4.037 ± 5.07 | 5.903 ± 7.187 | 12.974 ± 5.69 | 8.553 ± n.d | 26.069 ± 7.683 |

**(C) SIMPER Barren vs Developing (D) SIMPER Barren vs Mature (E) SIMPER Developing vs Mature**

| **Variable** | **Contribution (%)** | **Cumulative (%)** |  | **Variable** | **Contribution (%)** | **Cumulative (%)** |  | **Variable** | **Contribution (%)** | **Cumulative (%)** |
| --- | --- | --- | --- | --- | --- | --- | --- | --- | --- | --- |
| Mg exc | 11.28 | 11.28 |  | P available | 6.7 | 6.7 |  | pH | 10.93 | 10.93 |
| TOC/NTK | 9.61 | 20.89 |  | CO_2_/soil | 6.54 | 13.24 |  | NTK | 9.74 | 20.67 |
| DOC | 8.62 | 29.51 |  | CEC | 6.54 | 19.78 |  | K tot | 9.08 | 29.75 |
| Mn tot | 7.84 | 37.36 |  | TOC | 6.47 | 26.24 |  | CEC | 9.07 | 38.82 |
| K exc | 7.76 | 45.12 |  | Mg tot | 6.34 | 32.58 |  | Mn tot | 8.42 | 47.24 |
| CO_2_/C | 7.75 | 52.87 |  | K tot | 6.3 | 38.88 |  | TOC | 8.11 | 55.35 |
| DOC/TOC | 7.73 | 60.59 |  | DOC | 6.04 | 44.92 |  | CO_2_/soil | 6.16 | 61.51 |
| Na tot | 6.74 | 67.33 |  | NTK | 5.97 | 50.89 |  | Ca exc | 5.85 | 67.36 |
| P tot | 6.36 | 73.69 |  | Ca tot | 5.74 | 56.63 |  | Ca tot | 5.81 | 73.17 |
| Na exc | 5.38 | 79.07 |  | Na tot | 5.52 | 62.15 |  | P available | 5.22 | 78.39 |
| Mg tot | 5.29 | 84.36 |  | pH | 5.45 | 67.6 |  | Mg exc | 4.67 | 83.06 |
| Ca exc | 3.96 | 88.32 |  | DOC/TOC | 5.1 | 72.7 |  | Mg tot | 4.17 | 87.23 |
| Ca tot | 3.12 | 91.45 |  | CO_2_/C | 4.06 | 76.76 |  | P tot | 3.27 | 90.5 |
|  |  |  |  | K exc | 4.04 | 80.8 |  |  |  |  |
|  |  |  |  | Na exc | 3.96 | 84.76 |  |  |  |  |
|  |  |  |  | TOC/NTK | 3.89 | 88.65 |  |  |  |  |
|  |  |  |  | Ca exc | 3.77 | 92.42 |  |  |  |  |

**Supplementary Table S2. Pair-wise PERMANOVA for the 16S rRNA gene-based Illumina dataset**. Post-hoc test performed on the distance matrix generated according to OTU distribution along the Soil stage development (‘Barren’, ‘Developing’, ‘Mature’) bacterial communities (16S rRNA gene-based Illumina dataset) **(A)** within level 'Rhizosphere' and **(B)** within level 'Bulk soil'. Asterisk (*) indicates significant differences between samples.

| 1. **Groups** | **T** | ***P*** |
| --- | --- | --- |
| BARREN, DEVELOP | 1.3622 | 0.017* |
| BARREN, MATURE | 3.1865 | 0.001* |
| DEVELOP, MATURE | 2.7413 | 0.003* |

| 1. **Groups** | **T** | ***P*** |
| --- | --- | --- |
| BARREN, DEVELOP | 2.5847 | 0.001* |
| BARREN, MATURE | 5.3384 | 0.002* |
| DEVELOP, MATURE | 4.8999 | 0.002* |

**Supplementary Table S3. Comparisons of diversity indices among soil stages and fractions.** In the top two panels, mean values of diversity indices ± standard deviation are shown. In these panels, asterisks next to the metric’s name indicate significant differences in the median values (as per Kruskall-Wallis tests - * for *p*<0.05 and ** for *p*<0.01, respectively) across the different soil stages for the respective soil fractions (bulk soil or rhizosphere), while superscript letters indicate pairwise differences in the median values (as per Mann-Whittney pairwise tests) within each metric category. No asterisks or letters indicate non-significant differences. In the bottom panel, comparisons of the median values of each index between the two soil fractions at a given soil developmental stage (‘Barren’, ‘Developing’ and ‘Mature’) are shown (Kruskall-Wallis tests - * for *p*<0.05 and ** for *p*<0.01, respectively). R>B indicates higher values in the Rhizosphere fraction than in the bulk soil fraction, and vice versa for R<B. “n/s” stands for non-significant differences. All *p*-values were Bonferroni-corrected.

| Index | **Bulk soil** | | |
| --- | --- | --- | --- |
|  | ‘Barren’ | ‘Developing’ | ‘Mature’ |
| Shannon(log_2_)** | 7.917 ± 0.702^a^ | 8.539 ± 0.284^a^ | 9.07 ± 0.097^b^ |
| N. OTUs** | 2657 ± 546^a^ | 3418 ± 49^b^ | 2975 ± 91^a^ |
| Simpson** | 0.981 ± 0.012^a^ | 0.987 ± 0.002^a^ | 0.994 ± 0.001^b^ |
| Dominance** | 0.0189 ± 0.012^a^ | 0.013 ± 0.002^a^ | 0.0059 ± 0.0011^b^ |
|  |  |  |  |
| Index | **Rhizosphere** | | |
|  | ‘Barren’ | ‘Developing’ | ‘Mature’ |
| Shannon(log_2_)** | 8.726 ± 0.304^a^ | 8.751 ± 0.273^a^ | 9.401 ± 0.166^b^ |
| N. OTUs | 3062 ± 502 | 3152 ± 395 | 3691 ± 372 |
| Simpson | 0.989 ± 0.006 | 0.991 ± 0.003 | 0.995 ± 0.002 |
| Dominance | 0.0110 ± 0.0063 | 0.0089 ± 0.0029 | 0.0052 ± 0.0014 |
|  |  |  |  |
| Index | **Rhizosphere vs. Bulk soil** | | |
|  | ‘Barren’ | ‘Developing’ | ‘Mature’ |
| Shannon(log_2_) | R>B*** | n/s | R>B** |
| N. OTUs | n/s | n/s | R>B** |
| Simpson | n/s | n/s | n/s |
| Dominance | n/s | n/s | n/s |

**Supplementary Table S4**. **β-nearest taxon index (βNTI)**. βNTI values between each consecutive soil developmental stage (‘Barren’ to ‘Developing’ and ‘Developing’ to ‘Mature’) for each soil fraction (‘Bulk soil’ and ‘Rhizosphere’). The mean value ± standard deviation is reported.

| **Fraction** | **Soil developmental state** | |
| --- | --- | --- |
|  | **‘Barren’ to ‘Developing’** | **‘Developing’ to ‘Mature’** |
| **Bulk soil** | 9.62 ± 0.32 | 24.46 ± 0.52 |
| **Rhizosphere** | 7.69 ± 0.3 | 17.2 ± 0.84 |

**Supplementary Table S5. Relative abundance of the main bacterial** Phyla in the bulk soil and rhizosphere at each developmental stage (‘Barren’, ‘Developing’ and ‘Mature’).

| **Phylum** |  | **Bulk soil** |  |  |  | **Rhizosphere** |  |
| --- | --- | --- | --- | --- | --- | --- | --- |
|  | **‘Barren’** | **‘Developing’** | **‘Mature’** |  | **‘Barren’** | **‘Developing’** | **‘Mature’** |
| *Acidobacteria* | 6% | 6% | 10% |  | 6% | 14% | 14% |
| *Actinobacteria* | 21% | 24% | 23% |  | 12% | 14% | 22% |
| *Bacteroidetes* | 11% | 10% | 8% |  | 8% | 7% | 2% |
| *Chloroflexi* | 7% | 11% | 14% |  | 6% | 7% | 16% |
| *Cyanobacteria* | 8% | 7% | 1% |  | 26% | 4% | 0% |
| *Firmicutes* | 0% | 0% | 0% |  | 0% | 0% | 1% |
| *Gemmatimonadetes* | 1% | 1% | 1% |  | 1% | 1% | 2% |
| *Plantomycetes* | 4% | 4% | 5% |  | 2% | 2% | 2% |
| *Proteobacteria* | 31% | 28% | 24% |  | 32% | 44% | 24% |
| *TM7* | 4% | 4% | 3% |  | 2% | 1% | 3% |
| *Verrucomicrobia* | 4% | 3% | 5% |  | 5% | 4% | 7% |
| *Other* | 2% | 3% | 6% |  | 1% | 2% | 7% |

**Supplementary Table S6 (Excel file attached). LEfSe Analysis.** Linear discriminant analysis (Wilcoxon p-value: 0.05, LDA>2) Effect Size (LEfSe) of bacterial OTUs in the bulk soil and rhizosphere fractions in ‘Barren’, ‘Developing’ and ‘Mature’ soils.

**Supplementary Table S7 (Excel file attached). Network analysis.** Nodes and edge table for bulk soils and rhizospheres, at each soil developmental stage (‘Barren’, ‘Developing’ and ‘Mature’), are available on line in xlsx format.

**SUPPLEMENTARY FIGURES**

**Supplementary Figure S1. Principal Coordinates Analysis of bacterial community from the 16S rRNA gene-based PhyloChip dataset.** Principal Coordinates Analysis (performed on Bray-Curtis matrix on quantitative dataset) showing the clustering of bacterial communities according to soil fractions (bulk soils and rhizosphere; PERMANOVA, F_1,11_=2.6011; p=0.0345).


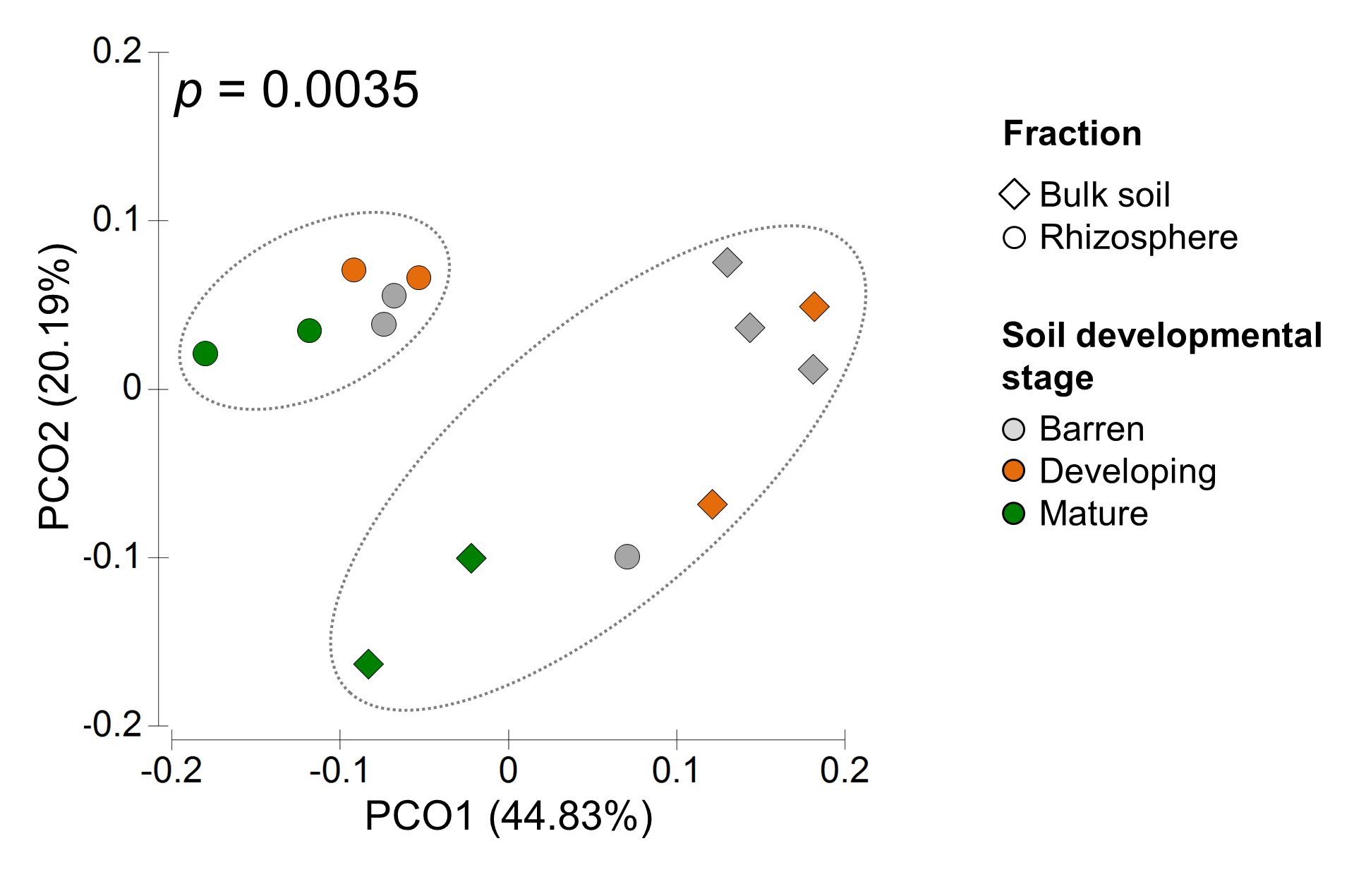


**Supplementary Figure S2. Principal Coordinates Analysis of bacterial community from 16S-23S rRNA ITS (internal-transcribed-spacer)-based ARISA dataset.** Principal Coordinates Analysis (performed on Bray-Curtis matrix on quantitative dataset) showing the clustering of bacterial communities according to soil fractions (bulk soil and rhizospheres; PERMANOVA, F_1,27_=2.7468; *p*=0.001).

**
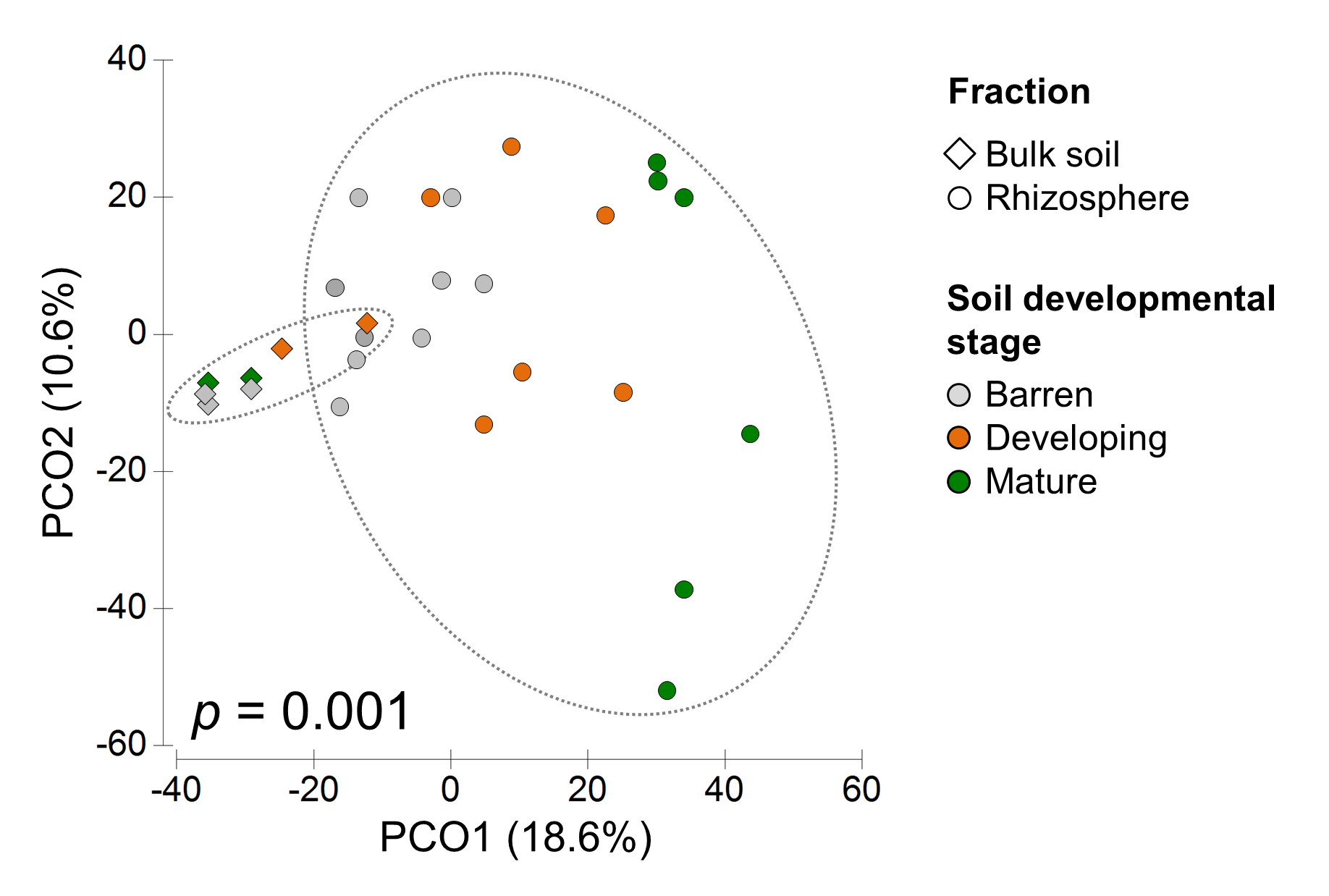
**

**Supplementary Figure S3.** Rarefaction and Goods’s coverage index

**
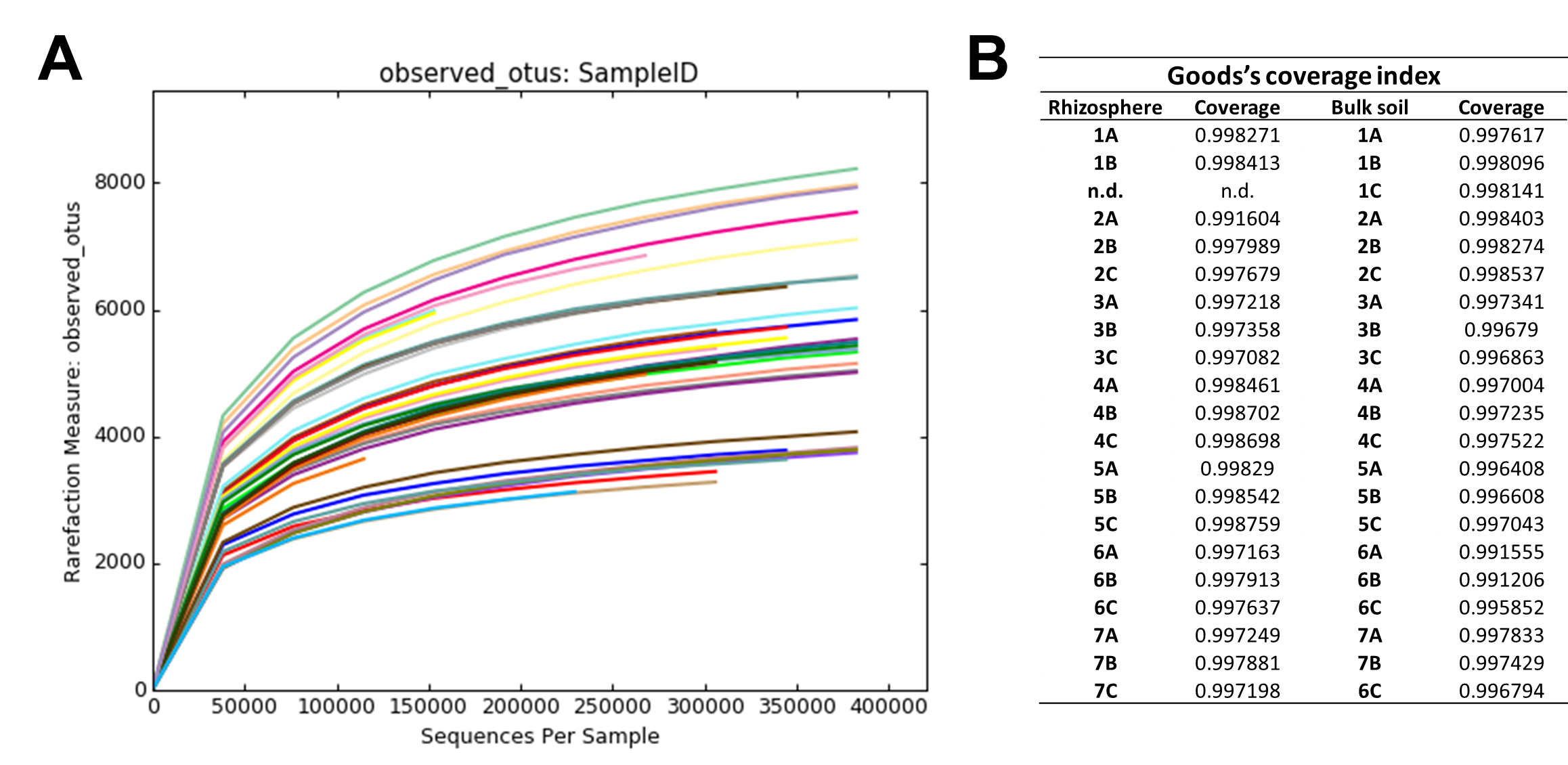
**

**Supplementary Figure S4. Bulk soils and rhizospheres bacterial community network topological parameters**. Total number of nodes **(A)** and total number of interactions and clustering coefficients **(B)** resulting from network analysis of bulk soils (red line) and Rhizospheres (black lines) across the three soils development stages ‘Barren’, ‘Developing’ and ‘Mature’.

**
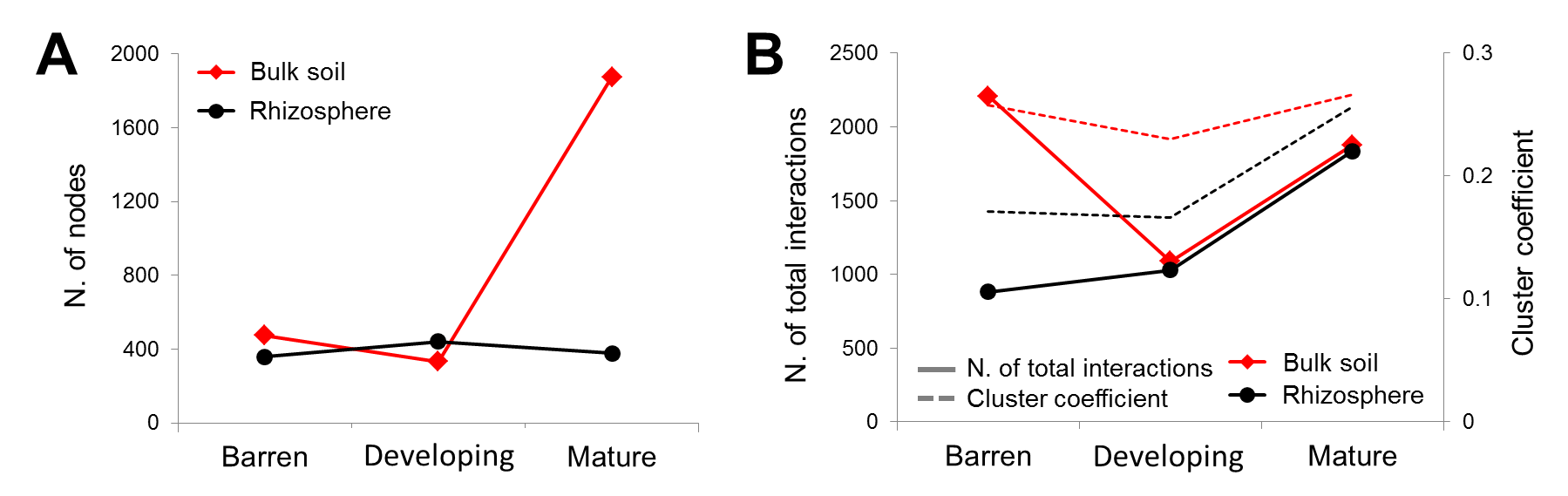
**

**Supplementary Figure S5. Topological coefficients, Betweenness Centrality and Node degree distribution of the co-occurrence network analysis showed in Fig. 6.** Red solid dots are values for the bulk soils network while blue dots are for the Rhizospheres. Red and Blues lines are the regressions of bulk soil and Rhizosphere values respectively.

**
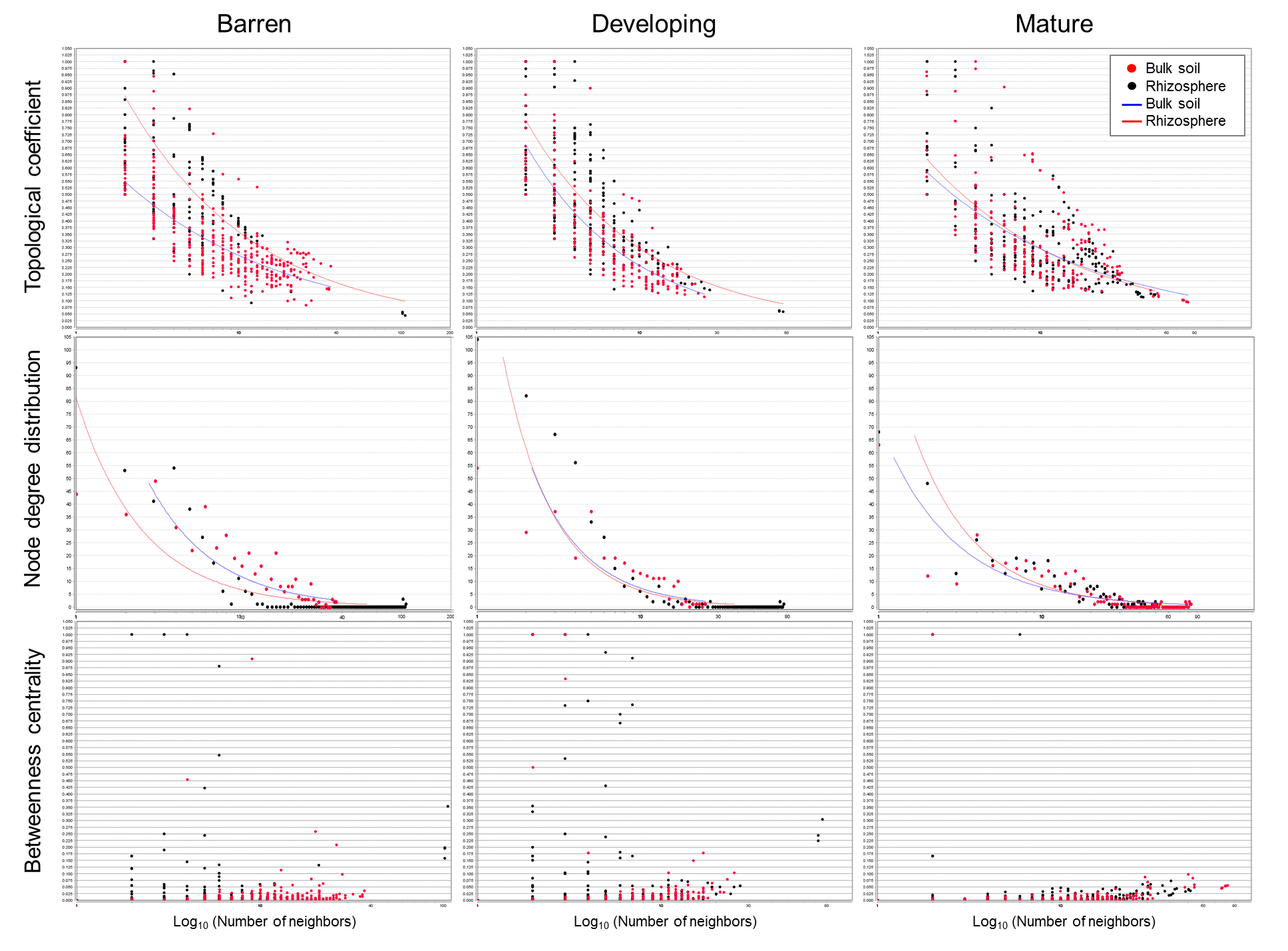
**

**SUPPLEMENTARY REFERENCE**

Anderson MJ. (2002). CAP: a FORTRAN computer program for canonical analysis of principal coordinates.

Anderson MMJ, Gorley RNRN, Clarke KR. (2008). PERMANOVA + for PRIMER: Guide to Software and Statistical Methods. In: Plymouth, UK.

Barberán A, Bates ST, Casamayor EO, Fierer N. (2012). Using network analysis to explore co-occurrence patterns in soil microbial communities. *ISME J* **6**: 343–351.

Bastian M, Heymann S, Jacomy M. (2009). Gephi: An Open Source Software for Exploring and Manipulating Networks. *Third Int AAAI Conf Weblogs Soc Media* 361–362.

Borin S, Ventura S, Tambone F, Mapelli F, Schubotz F, Brusetti L, *et al.* (2010). Rock weathering creates oases of life in a High Arctic desert. *Environ Microbiol* **12**: 293–303.

Brodie EL, DeSantis TZ, Joyner DC, Baek SM, Larsen JT, Andersen GL, *et al.* (2006). Application of a High-Density Oligonucleotide Microarray Approach To Study Bacterial Population Dynamics during Uranium Reduction and Reoxidation. *Appl Environ Microbiol* **72**: 6288–6298.

Caporaso JG, Kuczynski J, Stombaugh J, Bittinger K, Bushman FD, Costello EK, *et al.* (2010). correspondence QIIME allows analysis of high- throughput community sequencing data Intensity normalization improves color calling in SOLiD sequencing. *Nat Methods* **7**: 335–336.

Cardinale M, Brusetti L, Quatrini P, Borin S, Puglia AM, Rizzi A, *et al.* (2004). Comparison of different primer sets for use in automated ribosomal intergenic spacer analysis of complex bacterial communities. *Appl Environ Microbiol* **70**: 6147–56.

Doncheva NT, Assenov Y, Domingues FS, Albrecht M. (2012). Topological analysis and interactive visualization of biological networks and protein structures. *Nat Protoc* **7**: 670–685.

Edgar RC. (2010). Search and clustering orders of magnitude faster than BLAST. *Bioinformatics* **26**: 2460–2461.

Edgar RC. (2013). UPARSE: highly accurate OTU sequences from microbial amplicon reads. *Nat Methods* **10**: 996–998.

Faust K, Sathirapongsasuti JF, Izard J, Segata N, Gevers D, Raes J, *et al.* (2012). Microbial co-occurrence relationships in the Human Microbiome Ouzounis CA (ed). *PLoS Comput Biol* **8**: e1002606.

Klindworth A, Pruesse E, Schweer T, Peplies J, Quast C, Horn M, *et al.* (2013). Evaluation of general 16S ribosomal RNA gene PCR primers for classical and next-generation sequencing-based diversity studies. *Nucleic Acids Res* **41**: 1–11.

Konishi S, Kitagawa G. (2008). Information Criteria and Statistical Modeling. Springer New York: New York, NY.

Lozupone C, Lladser ME, Knights D, Stombaugh J, Knight R. (2011). UniFrac: an effective distance metric for microbial community comparison. *ISME J* **5**: 169–172.

Mapelli F, Varela MM, Barbato M, Alvariño R, Fusi M, Álvarez M, *et al.* (2013). Biogeography of planktonic bacterial communities across the whole Mediterranean Sea. *Ocean Sci* **9**: 585–595.

McDonald D, Price MN, Goodrich J, Nawrocki EP, DeSantis TZ, Probst A, *et al.* (2012). An improved Greengenes taxonomy with explicit ranks for ecological and evolutionary analyses of bacteria and archaea. *ISME J* **6**: 610–618.

Price MN, Dehal PS, Arkin AP. (2010). FastTree 2 – Approximately Maximum-Likelihood Trees for Large Alignments Poon AFY (ed). *PLoS One* **5**: e9490.

Scaglia B, Pognani M, Adani F. (2015). Evaluation of hormone-like activity of the dissolved organic matter fraction (DOM) of compost and digestate. *Sci Total Environ* **514**: 314–321.

Segata N, Izard J, Waldron L, Gevers D, Miropolsky L, Garrett WS, *et al.* (2011). Metagenomic biomarker discovery and explanation. *Genome Biol* **12**: R60.

Tsiamis G, Katsaveli K, Ntougias S, Kyrpides N, Andersen G, Piceno Y, *et al.* (2008). Prokaryotic community profiles at different operational stages of a Greek solar saltern. *Res Microbiol* **159**: 609–627.
